# Assessing patient perceptions of off-label cannabidiol use for insomnia through sentiment analysis

**DOI:** 10.1186/s42238-025-00306-7

**Published:** 2025-11-19

**Authors:** Gabriel Rodrigues Coutinho Pereira, Altobelli de Brito Mantuan, Julio Cesar dos Santos Oliveira, Gabriel Estevão Silva Fares, Vitor Manoel dos Santos Santiago Sá, Valéria Pereira de Sousa, Carlos Rangel Rodrigues, Lucio Mendes Cabral

**Affiliations:** 1 Faculty of Pharmacy, Federal University of Rio de Janeiro, 373 Carlos Chagas Filho Avenue, Rio de Janeiro, Rio de Janeiro, 21941-170 Brazil; 2 Computer Institute, Federal Fluminense University, Gen. Milton Tavares de Souza Avenue, Niteroi, Rio de Janeiro, 24210-310 Brazil

**Keywords:** Cannabidiol, Insomnia, Sentiment analysis

## Abstract

**Background:**

Recent global policy shifts have greatly expanded access to and the market for medicinal cannabis, broadening its availability for therapeutic use. These changes have led to a notable increase in off-label prescriptions of cannabidiol (CBD) based products, particularly for conditions such as epilepsy, anxiety, depression, insomnia, and chronic pain. Despite its growing popularity, clinical studies investigating the efficacy of CBD for insomnia remain limited, raising questions about its suitability for decision support. In this scenario, sentiment analysis provides an additional, low-cost, and measurable outcome of patients’ perceptions, which has proven valuable in offering an additional layer of understanding regarding the perceived effectiveness of treatments. Therefore, the objective of this study was to assess patients' perception regarding off-label CBD use for insomnia through sentiment analysis.

**Methods:**

English-language *tweets* related to CBD use for insomnia were collected from June 25, 2018, to January 9, 2023. The data was cleaned, and a representative subset of comments was manually labeled by experts. Then, a ROBERTA-based model was trained to automatically classify the remaining *tweets* in the database as either signals or noise. Finally, topic modeling and sentiment analysis were performed on the database of signal *tweets*.

**Results:**

From the 74,562 unique comments retrieved, 25,005 were classified as relevant based on both manual labeling and predictions made by the validated model. Topic modeling revealed eleven major themes, including the perceived efficacy of CBD for anxiety, pain, and insomnia, as well as practical considerations regarding treatment routines and preparation preferences. Sentiment analysis revealed positive feedback regarding CBD's use for insomnia, indicating that most users perceive it as an effective treatment for this condition.

**Conclusions:**

This study offers valuable insights into patient perceptions of off-label CBD use for insomnia, suggesting that CBD may indeed be beneficial for this condition, which aligns with existing, albeit limited, experimental evidence. Consequently, further research is necessary to confirm its efficacy. In this scenario, the application of sentiment analysis emerges as an effective tool for assessing patient perceptions, providing a richer context that complements the still limited evidence from traditional clinical trials on CBD use for insomnia.

Trial registration.

Not applicable.

**Supplementary Information:**

The online version contains supplementary material available at 10.1186/s42238-025-00306-7.

## Background

Phytocannabinoids are terpenophenolic compounds derived from *Cannabis spp.*, which include tetrahydrocannabinol (THC) – the primary agent responsible for the psychoactive effects of *Cannabis spp.* – and cannabidiol (CBD), the main non-psychotomimetic compound (Brucki, et al. 2021). Although *Cannabis sativa* is perhaps the most well-known species of the genus, it also encompasses other species, such as *Cannabis indica* and *Cannabis ruderalis*. Phytocannabinoids target the endocannabinoid (eCB) system, comprising receptors and their endogenous ligands, regulating neurotransmission in the central nervous system by modulating excitatory glutamate and inhibitory GABA signals. This system is widely distributed in the brain and plays a crucial role in cognition, motor function, sensory processing, reward, and emotions (Laksmidewi and Soejitno 2021).

Recent global policy changes have significantly expanded access to medicinal cannabis in over 50 countries. More than 33 states and the capital have already legalized its medicinal use in the United States, generating over a billion dollars in revenue in 2020 alone. This rise in market demand is closely linked to the 2018 U.S. Agriculture Improvement Act, which removed CBD products with less than 0.3% THC from the controlled substances list. Although the FDA has approved CBD for only a few specific applications, many individuals are able to obtain medical licenses for CBD products to treat a broader range of conditions beyond the approved on-label uses (Shannon et al. 2019; Sholler et al. 2020). This widespread adoption has contributed to North America’s dominance in the global cannabis market, accounting for approximately 80% of the market share in 2022. Globally, the cannabis market was valued at $43.72 billion in 2022 and is expected to grow at an impressive compound annual growth rate (CAGR) of 34.03%, reaching $444.34 billion by 2030 (Available from 2024).

The medicinal cannabis industry has grown considerably due to the therapeutic potential, safety, and non-psychoactive characteristics of CBD, which helps limit its recreational and improper use. There are also flourishing markets for other cannabidiol-based products, such as cosmetics, hair products, and pet products (Sholler et al. 2020). CBD has demonstrated anti-inflammatory, anticonvulsant, analgesic, anxiolytic, antipsychotic, and antidepressant effects (Brucki, et al. xxxx). These effects arise from its activity on various receptors in the nervous system and peripheral areas. Nonetheless, the strength of scientific evidence supporting each effect varies greatly, with some being well-supported and others requiring further investigation, as reviewed in depth elsewhere (Whiting et al. 2015; NASEM 2017). The complex pharmacology of CBD offers great therapeutic potential but also the potential for adverse effects and drug interactions, which must be considered and monitored (Huestis et al. 2019).

Epidiolex®, a direct derivative of cannabis that contains cannabidiol as its active ingredient, was approved by the FDA in 2018 for the treatment of refractory epilepsy in patients with tuberous sclerosis, Dravet syndrome, and Lennox-Gastaut syndrome. This decision paves the way for off-label prescriptions of the drug (Huestis et al. 2019), which have increased in recent years, particularly for treating other forms of epilepsy (Abu-Sawwa et al. 2020), anxiety, depression, insomnia, and chronic pain (VanDolah et al. 2019).

Nonetheless, the clinical trials conducted to investigate the efficacy of cannabidiol in treating insomnia still have limited scope and sampling, which may raise questions about their adequacy as support for decision-making (Huestis et al. 2019; Abu-Sawwa et al. 2020; VanDolah et al. 2019; Seltzer et al. 2020). In this context, analyzing patients’ perception through techniques such as sentiment analysis provides an additional, relatively low-cost and measurable outcome (Bulut and Poth 2022; Oliveira et al. 2019). Sentiment analysis involves the application of natural language processing (NLP) and machine learning techniques to extract subjective information from textual databases, thus allowing for the measurement of the intensity of opinions, *i.e.*, patient perception, related to a specific topic, product, or treatment (Reshi et al. 2022).

The popularization of social media platforms like Twitter (now X) generates a massive amount of textual data, facilitating the application of sentiment analysis, which has garnered attention in the business and healthcare sectors. The availability of data published on these platforms are sustained by users who express their opinions and impressions about a product or service, providing an overview of the researched topic (Giachanou and Crestani 2016). Approximately 40% of all health-related tweets reflect polarized emotions (*e.g.*, positive or negative), which can be quantified and serve as a basis for clinical decision-making (Gohil et al. 2018). Monitoring patient perceptions on social media can spontaneously reveal observations related to treatment routines and challenges, as well as reports of adverse events. Analyzing these comments can represent a wealth of relevant data for a trained specialist using the appropriate tools (Cocos et al. 2017; Lardon et al. 2018).

Thus, this study aims to investigate patient perceptions regarding the off-label use of CBD for insomnia by leveraging sentiment analysis to provide additional measurable outcomes that could assist clinical decision-making with improved cost efficiency. In doing so, this research seeks to develop a more comprehensive understanding of the subjective impacts experienced by users in managing insomnia. This focus is particularly relevant, as evaluating subjective aspects can reveal specific needs and challenges that may not be captured by objective measures, including therapy satisfaction and its influence on quality of life (Wankhade et al. 2022).

## Methods

### Data mining on twitter

#### Automated *tweets* extraction

The snscrape library (https://pypi.org/project/snscrape/) in Python was employed to extract *tweets* in English that included keywords related to the use of cannabidiol and phytocannabinoids for insomnia in different formulations. Specifically, *tweets* concerning the off-label use of cannabidiol for insomnia were automatically retrieved from the Twitter database from June 25, 2018, to January 9, 2023. This start date was established based on the approval of Epidiolex® by the FDA (Huestis et al. 2019).

The following combinations of keywords were employed in the Twitter searches conducted: “*cbd*” AND “*sleep*”; “*cbd*” AND “*insomnia*”; “*cannabidiol*” AND “*sleep*”; “*cannabidiol*” AND “*insomnia*”; “*canabidiol*” AND “*insomnia*”; “*canabidiol*” AND “*sleep*”; “*cbg*” AND “*insomnia*”; “*cbg*” AND “*sleep*”; “*cannabigerol*” AND “*insomnia*”; “*cannabigerol*” AND “*sleep*”; “*cannabis oil*” AND “*insomnia*”; “*cannabis oil*” AND “*sleep*”; “*cannabis spray*” AND “*insomnia*”; “*cannabis spray*” AND “*sleep*”; “*cannabis extract*” AND “*insomnia*”; “*cannabis extract*” AND “*sleep*”; “*cannabis product*” AND “*sleep*; “*cannabis product*” AND “*insomnia*”; “*sativa spray*” AND “*insomnia*; “*sativa spray*” AND “*sleep*”; “*sativa oil*” AND “*insomnia*”; “*sativa oil*” AND “*sleep*”; “*sativa extract*” AND “*insomnia*”; “*sativa extract*” AND “*sleep*”; “*indica spray*” AND “*insomnia*; “*indica spray*” AND “*sleep*”; “*indica oil*” AND “*insomnia*”; “*indica oil*” AND “*sleep*”; “*indica extract*” AND “*insomnia*”; “*indica extract*” AND “*sleep*”; “*ruderalis spray*” AND “*insomnia*”; “*ruderalis spray*” AND “*sleep*”; “*ruderalis oil*” AND “*insomnia*”; “*ruderalis oil*” AND “*sleep*”; “*ruderalis extract*” AND “*insomnia*”; “*ruderalis extract*” AND “*sleep*”; “*hemp oil*” AND “*insomnia*”; “*hemp oil*” AND “*sleep*”; “*hemp spray*” AND “*insomnia*”; “*hemp spray*” AND “*sleep*”; “*hemp extract*” AND “*insomnia*”; “*hemp extract*” AND “*sleep*”; “*epidiolex*” AND “*insomnia*”; “*epidiolex*” AND “*sleep*”; “*sativex*” AND “*insomnia*”; “*sativex*” AND “*sleep*”.

The raw data were stored as a Pandas DataFrame (McKinney and pandas, 2011) and underwent a cleaning process that involved removing *tweets* with character encoding issues (UTF-8 format), *retweets*, duplicate comments, and empty posts, *i.e.*, those containing only user mentions (@) or solely the search keyword, with or without a hashtag (#). To ensure anonymity and comply with best practices in privacy preservation, the authors of the posts and user mentions were converted into numerical identifiers and generic mentions (*i.e.*, @*twitter_user*).

To identify tweets containing personal opinions about the use of CBD for insomnia (signal) and to remove *tweets* containing noise, approximately 10% of the dataset were randomly sampled and manually labeled by two specialists. In cases of disagreement, a third specialist was consulted to determine the final classification of the *tweet*.

### Criteria used for classifying tweets

#### Signal class

The signal class included only tweets that contained clear and explicit personal experiences regarding the use of CBD for treating insomnia. This encompassed both pure and mixed formulations of CBD, including those combined with THC, CBG, CBN, or other cannabinoids. Various forms of cannabidiol and cannabis derivatives were considered, including extracts, sprays, oils, tinctures, gummies, and edibles.

As exclusion criteria for the signal class, *tweets* that could cause confusion regarding the pharmacological effects of cannabidiol for insomnia were removed. Therefore, we excluded comments explicitly mentioning the use of CBD in vaporizers or smoking, as well as mixed CBD preparations with melatonin, passionflower, chamomile, valerian, or the concurrent use of other drugs/compounds that could affect sleep quality, such as benzodiazepines, antipsychotics, analgesics, antihistamines, and antidepressants. Tweets mentioning CBD infusions and CBD teas were not considered, as the authors of these posts were likely referring to infusions of *Cannabis spp.*, leading to imprecise descriptions regarding the formulation used. Therefore, these *tweets* were classified as noise. Personal experiences with pure preparations of CBG, THC, or CBN were also excluded, as CBD would be present in very low concentrations.

The signal class was further sublabeled into subsets to enable a more detailed and stratified analysis of the tweets. These subsets were created to capture different dimensions of users' experiences and the context in which CBD was being used, ensuring that the data would support nuanced conclusions about CBD's role in treating insomnia. The subcategories included: “medicinal CBD”, “CBD dietary supplements”, “mixed CBD formulations”, “adverse events”, “dosage”, and “preparation types”, which allowed for a more refined investigation of users' reports.

Preparations containing pure or CBD-rich formulations with limited amounts of THC and other phytocannabinoids were included in the medicinal CBD subclass. This subclass covered products where CBD is the primary active compound, often found in regulated medical treatments. Therefore, tweets that explicitly described experiences with the use of CBD oils in different proportions were excluded, as well as preparations that could contain significant amounts of other phytocannabinoids, such as Sativex, full spectrum products, hemp oil, or oil from *Cannabis spp*. These formulations typically adhere to strict guidelines regarding the permissible levels of THC and other cannabinoids, making them suitable for therapeutic use without the psychoactive effects associated with higher concentrations of THC. By focusing on medicinal preparations, the analysis was able to target tweets that referenced the therapeutic application of CBD for insomnia, avoiding noise from mixed cannabinoid preparations or unregulated products. This distinction is critical for understanding the effects and experiences related specifically to medicinal CBD as a treatment for sleep disorders.

In addition, a subcategory related to CBD dietary supplements was established, which included tweets referencing gummies and edibles. Due to the lack of regulation, preparations such as CBD drinks, beverages, and CBD chocolates were excluded.

Mixed CBD formulations with other phytocannabinoids were also sublabeled. This subcategory included tweets that explicitly described personal experiences with oils like CBD:THC, CBD:CBG, CBD:CBN in varying proportions. Additionally, preparations that might contain significant amounts of other phytocannabinoids, such as Sativex®, full-spectrum products, hemp oil, and oils derived from *Cannabis spp.*, were included in this subclass.

The adverse event subset included comments mentioning adverse effects reported by users associated with CBD use, such as hepatotoxicity, appetite reduction, weight loss, gastrointestinal symptoms (diarrhea, vomiting, and nausea), irritability, drowsiness, agitation, insomnia, fever, rash (cutaneous hash), and anemia (Huestis et al. 2019).

#### Noise class

*Tweets* of a commercial nature, including advertisements from stores and sales suggestions directed at potential customers, were categorized as noise. This classification also encompassed *tweets* that shared scientific articles, research findings, newsletter, and information regarding the effects and benefits of CBD. Additionally, another prevalent type of noise included *tweets* that posed questions (e.g., “(…) CBD Rita’s….wonder if I’ll sleep good tonight…”) or offered vague usage recommendations, often with a commercial intent (e.g., “(…) CBD can relax your body and mind, and it will help you sleep through the night. Remember, you’ll need to take CBD consistently for at least two weeks to feel the effects.”). Tweets expressing extreme opinions, hate speech, or prejudice were also included in the Noise Class.

*Tweets* sharing third-party experiences (e.g., tweets describing the usage of CBD by friends or relatives) were considered noise to avoid duplication of reports. Preparations such as CBD tea and infusions were also categorized as noise due to their inaccurate descriptions of the formulations used. Preparations like bath salts (“salts,” “bath bombs”) or topical products (e.g., creams, balms, lotions, and salves) were included in the noise class, as they would not achieve systemic effects due to lack of absorption. Moreover, *tweets* describing combined administration of CBD with other non-cannabinoid drugs/compounds that could influence sleep quality—such as benzodiazepines, antipsychotics, analgesics, antihistamines, and antidepressants—as well as mixed formulations of CBD with melatonin, passionflower, chamomile, and valerian, were also classified as noise.

Additionally, tweets mentioning smoking (e.g., “cigarettes,” “dabs,” “joints”) and vaping (e.g., “vape,” “pen”) were classified as noise, as they are commonly associated with recreational cannabis use. Although smoking and vaping can also be part of medical cannabis use (Steinberg et al. 2022), these methods are more closely tied to recreational consumption, which may not reflect the therapeutic use of CBD. This distinction was made to avoid the potential biases introduced by recreational cannabis use, as medical and recreational cannabis users likely have different usage patterns, dosages, and health outcomes.

Smoking cannabis poses various health risks. For instance, the American Heart Association has raised concerns about the cardiovascular effects of smoking cannabis in both medicinal and recreational contexts. Additionally, low-strength evidence suggests links between long-term cannabis smoke and conditions, such as testicular cancer and chronic bronchitis. Findings for lung cancer and chronic obstructive pulmonary disease (COPD) remain inconclusive, though cannabis smoke contains many of the same carcinogens and mutagens found in tobacco smoke (Page et al. 2020).

Therefore, by excluding smoking and vaping routes, we aim to prioritize medically recommended methods of CBD administration, thereby improving the relevance and applicability of our findings to therapeutic contexts while minimizing confounding factors associated with recreational cannabis use.

## Model training and validation for signal detection

After manually classifying 10% of our tweet database into noise and signal, a machine-learning model was developed for the automated classification of the remaining data. The pre-trained ROBERTA model (Robustly Optimized BERT Pretraining Approach), an optimized version of the BERT model (Bidirectional Encoder Representations from Transformers), was selected for the construction of the noise and signal classification model (Liu et al. 1907). The manually labeled tweets underwent no text preprocessing, as the ROBERTA model was designed to work with raw text. The only modification applied to the tweet text was tokenization, which was carried out using the BERT tokenizer.

The ROBERTA-base model was chosen for training, which was carried out using the PyTorch library in Python (López-Zorrilla et al. 2023). Ten percent of the labeled tweets were randomly allocated to the test set using stratified sampling. The remaining 90% of manually labeled *tweets* were then split into training and validation sets. K-fold cross-validation, with folds ranging from 5 to 10, was evaluated, and the model yielding the highest F1-score on the test set was selected. The F1-score metric was selected as the decision parameter, as it is a harmonic mean of the precision and recall values. Therefore, it serves as a single performance measure that considers both false positives and false negatives (Giachanou and Crestani xxxx). A detailed description of the model architecture and training parameters is available in Additional file 1**.**

Model validation included evaluating predictive performance for the following metrics on the test set: accuracy, recall, precision, F1-score, and area under the ROC curve (receiver operating characteristic curve). The scikit-learn library was used for calculating the performance metrics of the generated models (Pedregosa et al. 2011). A confusion matrix for the model was also computed.

After the first validation round, the model's predictive performance was found to be insufficient for accurately classifying the remaining tweet dataset. The primary challenge stemmed from the significant class imbalance, as only 28% of the manually labeled sample belonged to the signal class, leading to suboptimal learning of the minority class. To mitigate this issue and improve model robustness, a sample balancing strategy was employed (Stanosheck et al. November 2023). Specifically, the model was first used to predict signal tweets from the unclassified portion of the dataset. From these, approximately 20% of the predicted signals were then manually reviewed and labeled according to the criteria previously described.

**Fig. 1:**
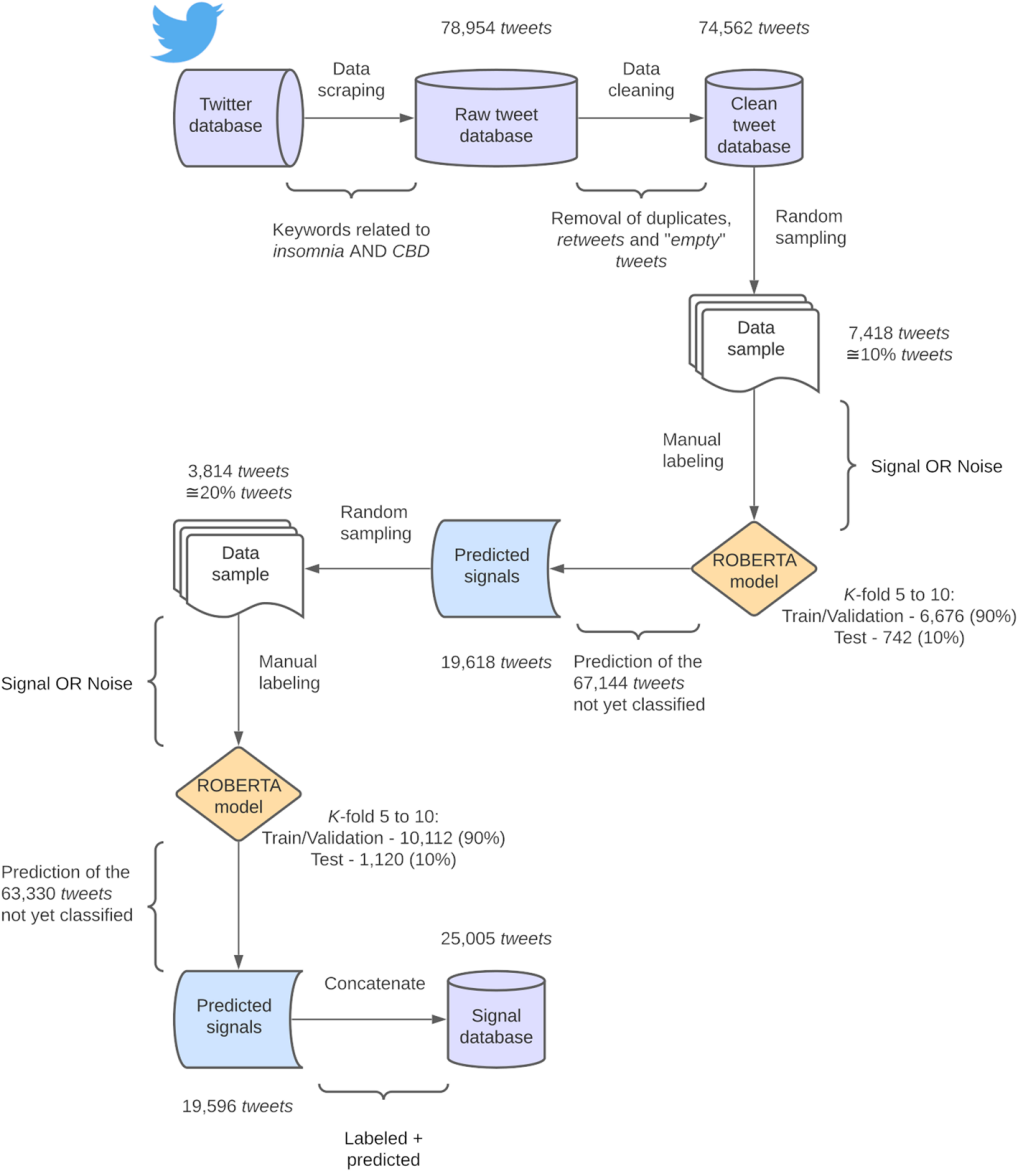
Schematic representation of the steps involved in constructing the database of signal *tweets*. Solid arrows represent the steps taken to construct the signal database, which includes both predicted and manually labeled signal tweets, containing self-reported experiences with CBD-containing products. The diagram was created using the Lucidchart server

This approach significantly improved sample balancing, ultimately increasing the proportion of signal tweets to 48%. The same training and validation process was applied to develop a second and more robust model. This model demonstrated significantly better performance compared to the initial one. It was then used to classify the remaining unclassified tweets. The training process outlined in this section is visually summarized in Fig. 1 aiming at a clearer understanding.

## Exploratory data analysis

### Topic analysis

All tweets included in the signal database, comprising both predicted tweets and manually classified ones, underwent pre-processing to enable subsequent exploratory data analyses. The pre-processing steps included: (I) tokenization; (II) conversion of text to lowercase; (III) removal of links (URLs and HTML); (IV) removal of user mentions, numbers, and punctuation (including hashtags); (V) removal of stopwords; (VI) lemmatization; and (VII) Part-of-Speech (POS) tagging.

Tokenization was performed using the TweetTokenizer function from NLTK library in Python (Bird et al. 2009). The Pipeline function from the stanza library was utilized for lemmatizing the text of the tweets (Qi et al. 2020). Regular expressions were used to remove URLs, HTML, user mentions, numbers, punctuation, and hashtags. The tweets were converted to lowercase, and stopwords were removed using the stopwords function from the NLTK library (Bird et al. 2009).

The text was additionally processed using POS tagging available in the NLTK library in Python (Bird et al. 2009), which allows for the identification of grammatical classes in sentences. Only the following English grammatical classes, the native language of the tweets, were considered for subsequent analyses: noun (NN), verb base form (VB), past tense verb (VBD), gerund or present participle verb (VBG), past participle verb (VBN), and third-person singular present verb (VBZ).

The processed tweet texts were subsequently used to compute the TF-IDF (Term Frequency-Inverse Document Frequency) descriptor. We then performed topic analysis using the Latent Dirichlet Allocation (LDA) method with the Gensim library to identify the main themes present in the tweet database (Min et al. 2019). To determine the optimal number of topics, denoted as K, we evaluated the model's coherence score for values ranging from 5 to 20, utilizing both unigrams and bigrams. We selected a λ value of 0.6 to regularize the weight of the word distributions for each topic (Min et al. 2020).

The results of the topic analysis were interpreted using the interactive tool PylDavis, which visually displays the distances between topics (Egger and Yu 2022).

To further explore the contextual nuances and semantic relationships within the dataset, we extended our analysis by employing BERTopic, a state-of-the-art large-language modeling approach for topic modeling. BERTopic leverages transformer-based embeddings to capture the subtle meanings and connections within the text, thereby providing additional insights into the data (Grootendorst and BERTopic 2022). The "all-MiniLM-L6-v2" embedding model, available in BERTopic, was initially employed to convert documents into high-dimensional bigram vectors. Dimensionality reduction was achieved using the UMAP (Uniform Manifold Approximation and Projection) algorithm, ensuring the preservation of the data's underlying structure in a lower-dimensional space. Unsupervised clustering was then performed using HDBSCAN (Hierarchical Density-Based Spatial Clustering of Applications with Noise). Finally, the KeyBERTInspired representation model was selected to extract representative terms for each topic based on class-based TF-IDF (c-TF-IDF) scores (Grootendorst and BERTopic 2022).

### Manual grouping of tweets

After conducting the topic analysis, we manually grouped comments related to formulation (subcategory 1) and/or concentration (subcategory 2) to better understand the preferences and usage patterns of CBD for insomnia within the online community. Additionally, we mapped the primary adverse events reported as related to CBD (subcategory 3), which offered valuable insights into the compound's safety across different usage contexts. Due to the large volume of comments predicted as signals in our database (Fig. 1), the manual grouping of tweets was limited to those manually labeled.

**Fig. 2:**
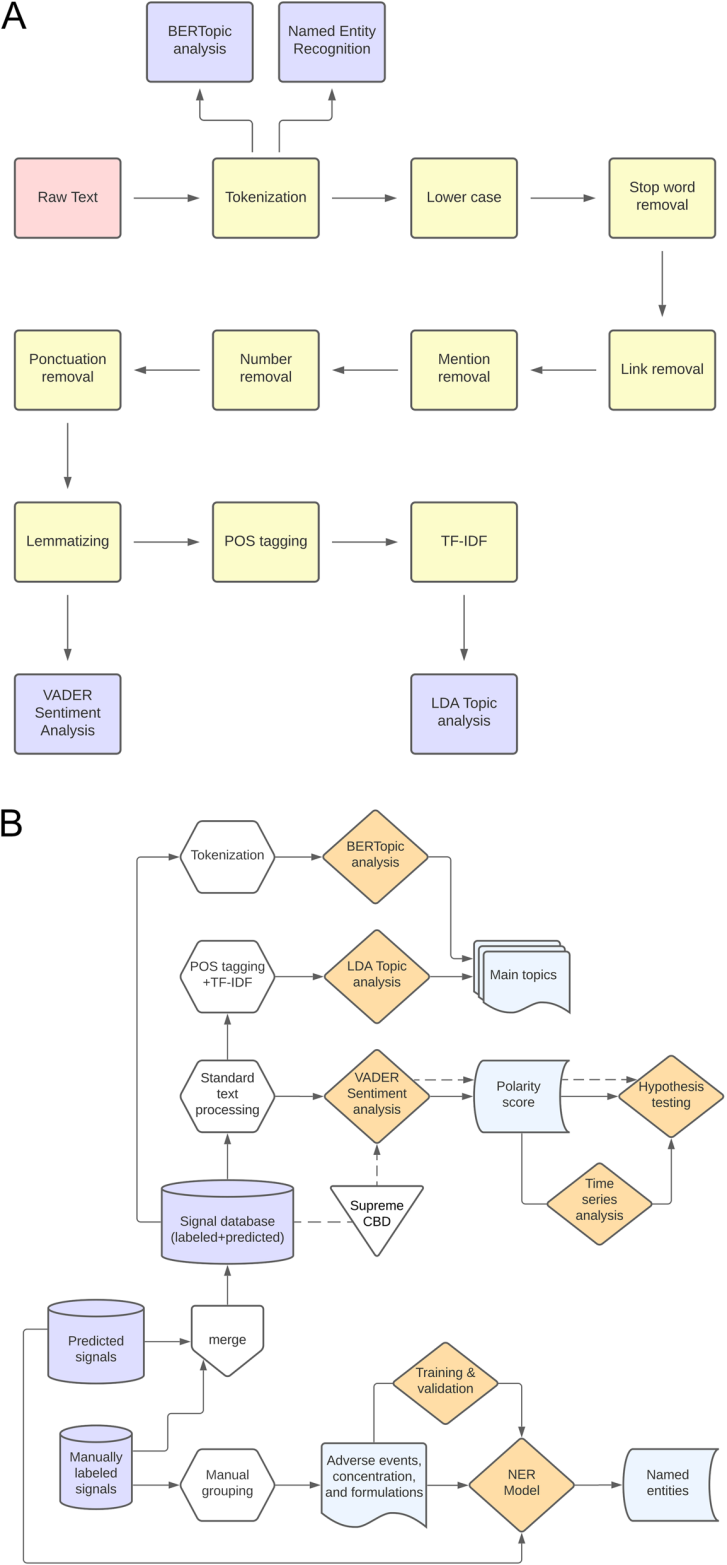
Steps involved in exploratory data analysis and patient perception analysis. **A** Text processing of tweets. **B** The workflow of exploratory and sentiment analyses. Solid arrows represent the steps conducted for the signal database containing self-reported experiences with CBD-containing products, while dashed arrows represent the steps conducted for the database containing only descriptions of SupremeCBD oil. The diagram shown in the figure was created using the Lucidchart server

Through this analysis, we identified a substantial number of comments mentioning CBD oil products from the brand SupremeCBD. This led us to search for user-reported experiences with SupremeCBD oil formulations by using the keyword “supreme” in our entire signal database, which comprises both manually labeled and predicted entries (Fig. 2). Following this keyword search, experts reviewed and confirmed the presence of these formulations in the selected tweets.

### Named entity recognition

Named-entity recognition (NER) is a fundamental NLP task focused on identifying and categorizing entities such as names, organizations, locations, and domain-specific terms (e.g., medical conditions or product names). Recent advancements in large language models (LLMs) have significantly enhanced NER performance by effectively capturing context-dependent word relationships. This enables LLMs to accurately recognize complex, multi-token entities and makes them a powerful tool for domain-specific NER challenges (Perera et al. 2020).

In this context, we employed the Mistral-7B-Instruct-v0.2 model for NER to identify key entities within the predicted signal tweets, providing a complementary and systematic extension to our prior manual analysis. To achieve this, three distinct NER models were fine-tuned to detect specific entities across the following categories: CBD formulation (subcategory 1), CBD concentration (subcategory 2), and adverse events associated with CBD use (subcategory 3).

For fine-tuning Mistral-7B-Instruct-v0.2 model, 90% of the labeled tweets from the signal database (5.408 tweets) were allocated to the training process, with 80% of this subset used for training and the remaining 20% reserved for validation (evaluation). The remaining 10% of the labeled tweets, comprising unseen data, were allocated to the test set. Notably, the manually labeled tweets underwent no text preprocessing, as Mistral were specifically designed to work with raw text.

Mistral-7B-Instruct-v0.2 is a 7-billion-parameter decoder-only transformer model developed by Mistral AI, pre-trained on a diverse corpus and specifically optimized for text generation tasks. Leveraging advanced mechanisms such as Grouped Query Attention (GQA) and Sliding Window Attention (SWA), it improves context handling and reduces computational load. This combination allows for competitive and efficient performance compared to other available LLMs, making it well-suited for a wide range of NLP tasks, including NER and sentiment analysis (Hu et al. 2021).

The SentencePiece Byte-Pair Encoding, compatible with the Mistral-7B-Instruct-v0.2 model, was selected for text tokenization. The pretrained model was loaded with 4-bit quantization to optimize computational efficiency. Training was conducted for a total of 4 epochs with a learning rate of 4 × 10^–4^. Gradient accumulation was set to 2 steps, and a linear learning rate scheduler was used with 20 warmup steps. The optimizer used was paged_adamw_8bit. LoRA (Low-Rank Adaptation) was applied to improve training efficiency, with the parameters lora_alpha = 16, lora_dropout = 0.05, and *r* = 16. The model was deployed for NER tasks using the generate function, with specific settings: temperature of 0.2, top-p sampling of 1.0, and top-k set to 10.

Training and evaluation were managed using the SFTTrainer framework, with evaluation conducted at the end of each epoch based on the eval_loss metric. The process incorporated the following prompts as an instruction to each model:

- Subcategory 1: “*You are an expert linguist specializing in cannabidiol (CBD) and cannabis-derived products. Your task is to perform Named Entity Recognition (NER) on the provided text, specifically identifying entities tagged as "CBD_PRODUCT" that are self-reported as being used in the context of insomnia. This tag should include explicit mentions of CBD or cannabis-derived products, such as "CBD gummy," "CBD spray," "CBD oil," "hemp oil," and "cannabis oil." Focus on capturing product mentions within the context of insomnia-related discussions.*”
- Subcategory 2: “*You are an expert linguist specializing in cannabidiol (CBD) and cannabis-derived products. Your task is to perform Named Entity Recognition (NER) on the provided text, specifically identifying entities tagged as "CBD_COMPOSITION" that include either dosage information (e.g., "50-70 mg CBD," "2-4 mg THC") or drug composition (e.g., "1:2 CBD to THC"). Focus on capturing mentions of CBD or cannabis-derived products with dosage or composition details, particularly in the context of insomnia-related discussions.*”
- Subcategory 3: “*You are an expert linguist specializing in cannabidiol (CBD) and cannabis-derived products. Your task is to perform Named Entity Recognition (NER) on the provided text, specifically identifying entities tagged as "CBD_ADVERSE_EVENT" that refer exclusively and explicitly to adverse events associated with the use of CBD. Examples of such adverse events include, but are not limited to, "nightmare," "tiredness," "sleep paralysis," "lethargy," "intoxication," "dizziness," "irritation," "grogginess," "tinnitus," "drowsiness" and other similar terms. Focus on capturing mentions of adverse effects explicitly attributed to CBD, particularly in the context of insomnia-related discussions.*”

The validation of the NER models was conducted by assessing their predictive performance on the test set using the following metrics: accuracy, precision, recall, and F1-score (Pedregosa et al. 2011). After validation, we applied automated NER exclusively to the predicted signal database, as the manually curated signal database of 5,408 tweets had been used to fine-tune and validate these models (Fig. 2).

## Analysis of patients’ perception

The perceptions of CBD users regarding insomnia were investigated through sentiment analysis of signal tweets using the VADER method (Valence Aware Dictionary and sEntiment Reasoner). VADER is a lexicon-based method that has shown superior performance compared to individual human evaluations in a benchmark task involving English-language tweets (Hutto and Gilbert 2014). The algorithm outputs a sentiment score, or compound score, reflecting the polarity and intensity of the sentiment expressed in a post.

Then, the raw text from the signal tweets was processed only up to the lemmatization step to retain adjectives and adverbs, which is fundamental for sentiment analysis (Hutto and Gilbert 2014). The flowchart presented in Fig. 2 illustrates the steps involved in the text processing for this study.

The sentiment scores predicted by VADER were used to assess patient satisfaction with CBD for insomnia, as detailed in personal experience reports within the complete database of signal tweets (group 1). Additionally, we specifically examined personal experiences related to the use of CBD oil from the SupremeCBD brand (group 2), which contains less than 0.2% THC, in compliance with European Union regulations. The EU enforces strict standards for the marketing of CBD products to ensure consumer safety and adherence to public health regulations (Silva and O e, Figueiredo EN. 2023).

Initially, the normality of the sentiment scores for both groups (group 1 and group 2) was assessed using the Shapiro–Wilk test (*p*-value < 0.05), available in the SciPy package in Python (Virtanen et al. 2020). This analysis aimed to inform the selection of the most appropriate statistical test for subsequent comparisons.

Once the non-normality of the groups was established, we applied the non-parametric Wilcoxon signed-rank test to compare the median of each group against a hypothetical distribution with a median equal to zero. The alternative hypothesis considered was that the median of the signal database is statistically greater than zero, with a 95% confidence level (Rosner et al. 2006). This analysis was conducted to assess whether patients provided favorable feedback regarding the use of CBD for insomnia. The SciPy library in Python was used for this task (Virtanen et al., 2020).

The sentiment scores obtained from the VADER method were aggregated monthly, enabling the calculation of mean sentiment and facilitating the assessment of monthly trends. From the aggregated monthly time series data, we employed the Phillips-Perron and Augmented Dickey–Fuller statistical tests, available through the arch and statsmodels libraries in Python, to check for stationarity in the data (Wong and Nguyen 2022; Silva et al. 2021). Since the first and last months of the sample were incomplete, they were removed from the time series for better comparison. The goal of this analysis is to determine whether patients' perceptions of CBD for insomnia were influenced by the approval of Epidiolex. At this stage, we did not evaluate the group of tweets containing reports of SupremeCBD (group 2), as the company registered its trademark only on December 18, 2019 (Registration Nº. 12,370,423), beginning to market its products, including CBD oil, only after that period.

Aiming at a better understanding of the steps involved in exploratory data analysis and patient perception analysis, a flowchart is provided in Fig. 2.

## Results

### Data mining on twitter

The search for tweets related to the use of CBD for insomnia yielded 78,954 tweets. After eliminating duplicates, retweets, "empty" tweets, and comments with character encoding issues, 74,562 unique comments remained in the dataset (Additional file 2).

When manually classifying an initial random sample of 7,418 tweets—about 10% of the total dataset—we found that it was imbalanced, with only 28% identified as signals and 72% as noise. This suggests that a large portion of the extracted tweets were purely commercial, which adds noise to the sentiment analysis. Thus, it was essential to develop a model that could effectively distinguish between commercial tweets—those unrelated to the study's objectives—and tweets that genuinely provided insights into user experiences with CBD concerning insomnia and sleep outcomes.

The tweets manually classified as noise were mostly commercial, including advertisements from stores and suggestions from sellers to potential customers. Additionally, tweets promoting scientific articles, presenting research results, and providing information about the effects and benefits of CBD were also frequent. Representative examples of the noise and signal classes are presented in Table 1.

**Table 1:** Representative *tweets* for each class

Class	Representative *Tweets*
Signal	*•* *I’ve been taking CBD to help me sleep and honestly it works wonders #cbd* *•* *@twitter_user I take it to help me sleep (I sometimes have insomnia spells). Edison CBD Oil, I think the dosage is 10 mg per ml* *•* *@twitter_user Hey if it helps, i personally take 10 mg timed release (from iherb) and they helped me so much with my sleep. My cbd/thc helps too but they’re medicinal so probably different strains than what you have* *•* *I ate some CBD edibles to help me sleep. I’m still wide awake* *•* *@twitter_user Your body natural produces melatonin. Its okay every now and then but not daily. Try cbd oil or gummies. Its done wonders for me. I sleep so good!* *•* *@twitter_user @twitter_user Because I don’t get restorative sleep with having fibromyalgia—CBD actually has improved my sleep quality and thus I can go to work and function properly without the hangover of prescription medication*
Noise	*•* *@ twitter_user @ twitter_user Also CBN is gonna make you more sleepy CBD will help with the anxiety. Really depends on the terpene profile but if you find something high in CBN that will 100% be better for sleep* *• Anyone here experienced with #CBD? Is it really effective to regulate sleep?* *•* *@twitter_user @twitter_user Yeah, except I vape a high CBD/ THC mix. That'll get me about an hour of unfragmented sleep before the leg nerves start acting up, and I start kicking myself awake every couple of minutes* *•* *CBD Oil 500 mg Wholesale pricing $59.97 50 servings— *https://external_URL* #pain #insomnia #anxiety #depression #ptsd #Seizures #focus #stress *https://external_URL *•* *@ twitter_user Have you ever tried CBD for sleep? We're giving away $500 worth of CBD today! Check out our latest tweet.* *•* *Kent Reporter: Best CBD Oil for Sleep and Insomnia in…. *https://external_URL via* @twitter_user* • *A recent study of people suffering from Parkinson's disease indicated that the cannabinoid was able to reduce REM (rapid eye movement) sleep disorders. A 2019 sleep quality study found that more patients experienced improved sleep than disrupted sleep when using CBD.#zellefarms*

## Model training and validation for signal detection

The model that achieved the highest F1-score was k-fold 8, with a value of 0.81. Nonetheless, this indicated that the model's predictive performance was not yet robust enough to classify the remaining tweets in the study context. Given that, only 28% of the manually classified sample consisted of tweets from the signal class—indicating an imbalanced dataset—a sample balancing strategy was employed to enhance the model's robustness (Stanosheck et al. November 2023). This strategy involved predicting the labels for the remaining unlabeled tweets using the model mentioned earlier and manually verifying about 20% of the classified tweets as signals. Considering that the k-fold 8 model attained an F1-score of 0.81, which substantially outperforms a random model, this approach can potentially enhance the identification of tweets belonging to the signal class compared to those representing noise.

The k-fold 8 model classified 67,144 unlabeled tweets, predicting a total of 19,618 signals. From these, 3,914 tweets (approximately 20%) were manually labeled according to the same criteria previously established. This approach successfully balanced the sample, leading to the identification of 3,297 new signals, resulting in a final distribution of 48% signals and 52% noise. The same training and validation procedures were applied to develop a second model, referred to as the final model, which proved to be significantly more robust than the first one, as outlined below.

Following the second and final round of training, the model demonstrating the highest predictive performance was the one trained using k-fold 10, achieving a substantially higher F1-score of 0.90. This model was designated as the reference model for our study. The probability threshold for class separation was set at 0.38, which corresponds to the value that optimizes the F1-score metric.

**Fig. 3:**
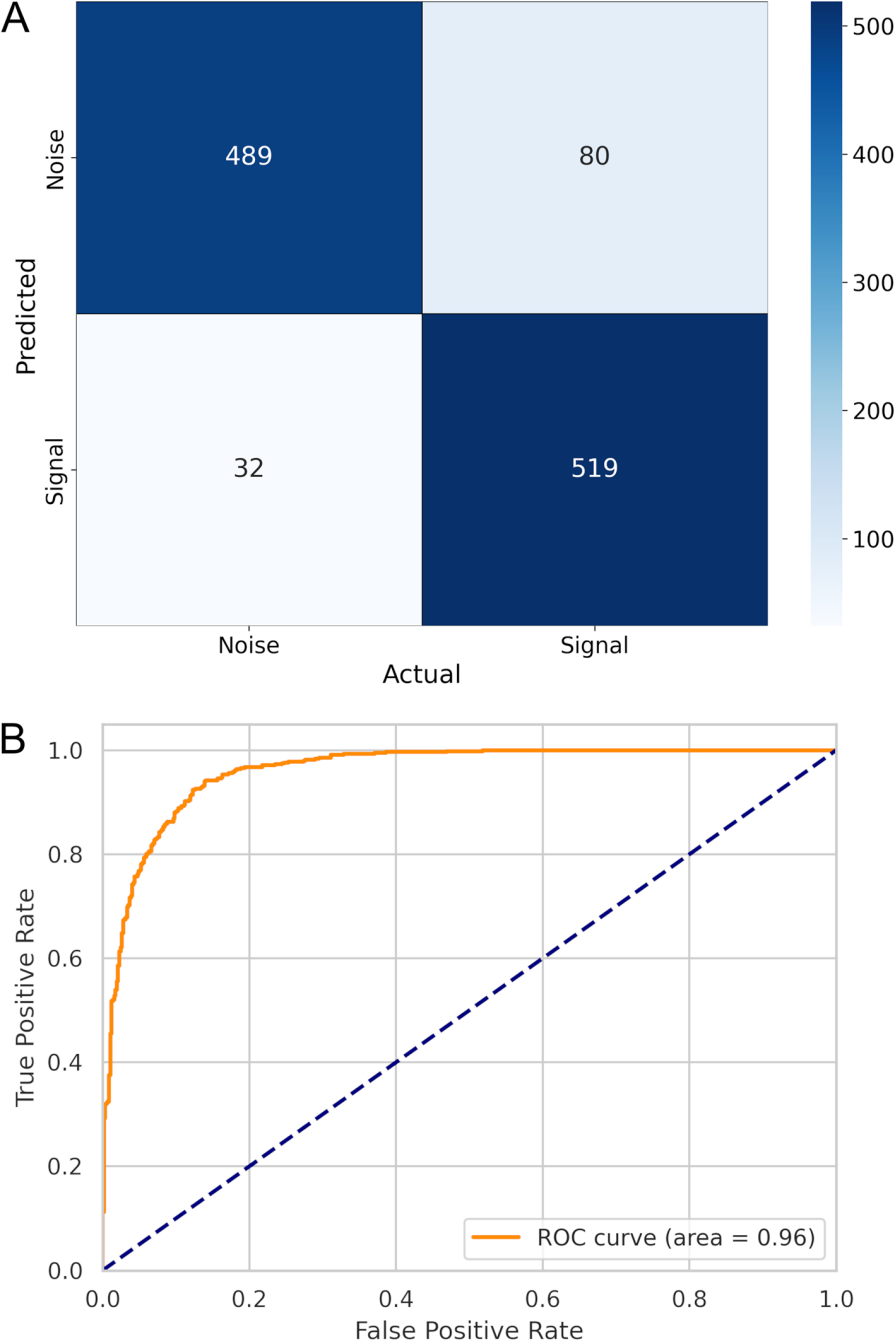
Predictive performance metrics calculated for classifying signal and noise tweets using the fine-tuned RoBERTa model (with tenfold cross validation) on the test set. **A** Confusion matrix showing the binary classification performance of the fine-tuned model. **B** Receiver Operating Characteristic (ROC) curve representing the model’s performance across all thresholds. The solid orange line represents the performance achieved by the fine-tuned model, as indicated by the area under the ROC curve (AUC-ROC) of 0.96. The dashed blue line indicates the performance of a random model, corresponding to an AUC-ROC of 0.5

As displayed in the confusion matrix (Fig. 3A), the final model correctly classified 93.8% of the noise (True Negative) and 86.7% of the signals (True Positive) in the test set. Nonetheless, it misclassified 13.3% of the signals as noise (False Negative) and only 6.1% of the noise as signals (False Positive). Furthermore, it demonstrated an AUC-ROC value of 0.96 (Fig. 3B), indicating its efficiency in distinguishing between observations of the noise and signal classes (Hajian-Tilaki 2013).

The model also achieved an accuracy of 90% on all observations in the test set, which was unseen by the algorithm, demonstrating its strong ability to generalize to new data. Among all positive predictions, 86% are true positives, as indicated by the precision metric. Together with the recall metric, which shows that 94% of all true positive values were correctly identified within the test set, we can conclude that the model demonstrated a high level of confidence in predicting the signal class. The F1-score value, which is a harmonic mean between precision and recall, further supports this conclusion (0.90).

After validation, the model was employed to classify the remaining unlabeled tweets as either noise or signal. The tweets predicted as signals underwent manual inspection by specialists, who removed any false positives from the dataset. The final database, which included both manually labeled tweets and those predicted by the model, comprised a total of 25,005 signals (Fig. 1).

## Exploratory data analysis

### Topic analysis

**Fig. 4:**
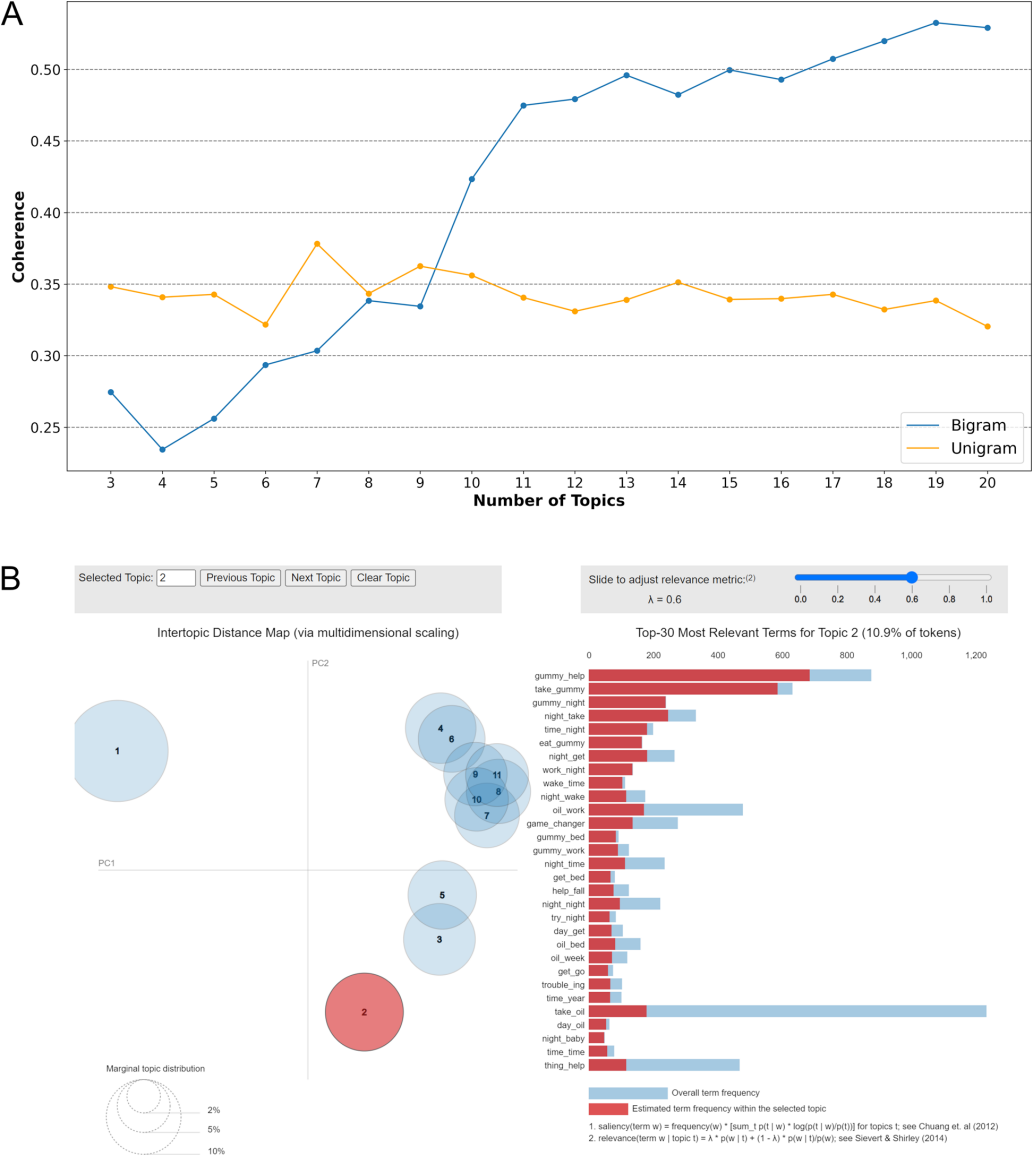
Latent Dirichlet Allocation (LDA) topic analysis on Twitter comments on cannabidiol use for insomnia. **A** Coherence score values for the LDA models investigated with *k* ranging from 5 to 20. The scores computed for both unigrams (orange) and bigrams (blue) are displayed as dotted lines. **B** Visual inspection of the LDA results for topic 2 (CBD as a supplement/food) on the pyLDAvis platform. The top 30 most relevant bigram terms for Topic 2 are displayed as a side bar plot, showing the estimated term frequency within the selected topic (red) compared to the overall term frequency (blue-gray), with k set to 11 topics and a relevance metric (λ) of 0.6

To select hyperparameters that optimize the model's coherence score, we explored a grid of k topics ranging from 5 to 20, using both unigrams and bigrams (Fig. 4). The coherence score metric measures the degree of similarity between the keywords within the same topic, which is proportional to the interpretability and quality of the topics identified by the model (Min et al. 2020). This analysis suggests that, overall, the model with bigram tokens offered better data interpretability, given its higher coherence score values. Additionally, the k value of 11 topics on the coherence score curve for bigrams corresponds to an inflection point, indicating a sharp reduction in the gain of the coherence score as more k topics are added. Therefore, following the elbow method, the bigram model with k = 11 topics (coherence score = 0.47) offered optimal interpretability (Kim et al. 2023) and was subsequently selected for the topic analysis.

The topics were categorized into eleven themes based on the graphical analysis of the LDA, as shown in Fig. 4, and the authors' interpretation of the keywords (Table 2). The representative themes of the dataset include perceived efficacy of CBD for anxiety (topic 1), CBD as a supplement/food product (topic 2), treatment routine (topic 3), transformative experiences with CBD (topic 4), nighttime use of CBD (topic 5), perceived efficacy of CBD for joint pain (topic 6), CBD concentration in products (topic 7), general benefits of CBD (topic 8), CBD oil (topic 9), perceived efficacy of CBD for insomnia (topic 10), and perceived efficacy of CBD for chronic pain (topic 11). The key aspects addressed in each topic were also detailed in Table 2.

**Table 2:** Topics Identified in the LDA Analysis

Topic	Keywords (Bigrams)	Description
1. Perceived efficacy of CBD for anxiety	*oil_help, help_anxiety, help_relax, oil_anxiety, anxiety_depression, anxiety_help*	Discussions focus on how CBD can be effective in treating anxiety, highlighting personal experiences and user reports of perceived benefits
2. CBD supplement/food	*gummy_help, take_gummy, gummy_night, eat_gummy, gummy_bed, gummy_work*	Comments center on CBD as a supplement or food, particularly in the preparation of gummies
3. Treatment routine	*get_night, take_bed, use_product, hour_take, take_half, couple_hour*	Users share their CBD treatment routines, preferred administration times, preparation methods, and favored forms of use
4. Transformative experiences with CBD	*change_life, help_good, work_wonder, oil_change, help_anything, thank_god*	Comments in this topic report personal transformative experiences using CBD, including improvements in physical and mental health
5. Nighttime use of CBD	*oil_night, night_month, night_help, get_night, week_night, oil_time*	Users discuss the use of CBD at night, focusing on how it can aid in relaxation and improve sleep quality when administered during this time
6. Perceived efficacy of CBD for joint pain	*joint_pain, help_joint, help_pain, know_work, help_deal, thing_work*	Explores how CBD may be effective in relieving joint pain and inflammation, providing relief for those suffering from these conditions
7. CBD concentration in products	*take_mg, mg_mg, dose_help, try_mg, mg_night, mg_gummy*	Discussions focus on the concentration and presentation of CBD-based products, with users sharing their experiences and consumption recommendations
8. General benefits of CBD	*improve_anxiety, help_headache, help_arthritis, help_lack, relax_muscle, help_shoulder*	Comments address the general benefits of CBD for various pathophysiological conditions, such as anxiolytic, anti-inflammatory, and antinociceptive effects
9. CBD oil	*oil_yesterday, oil_tongue, order_oil, person_oil, put_oil, oil_way*	This topic specifically focuses on CBD oil, discussing its composition, extraction methods, quality, and common uses
10. Perceived efficacy of CBD for insomnia	*help_dream, bed_baby, night_amazing, oil_knock, oil_bed, bed_night*	Users share their experiences using CBD for treating insomnia, highlighting how it can help improve sleep quality and reduce sleep issues
11. Perceived efficacy of CBD for chronic pain	*chronic_pain, help_knee, life_changer, pain_relax, knee_pain, help_chronic*	Lastly, this theme addresses the use of CBD for chronic pain relief, exploring its effectiveness as an alternative to traditional medications

We then expanded our analysis by incorporating BERTopic, a topic modeling algorithm based on large language models, to better capture contextual relationships within the data (Grootendorst and BERTopic 2022). Notably, to enable a fair comparison with the results obtained from LDA and the distribution of topics, we focused on the top 11 topics ranked by the number of tweets, as detailed in Additional File 3. This approach allowed us to assess how the semantic granularity of BERTopic aligns with or deviates from the themes uncovered by probabilistic models such as LDA, providing a thorough perspective on the dataset.

The top 11 topics were categorized based on the authors' interpretation of the representative bigram keywords and illustrative tweets, as outlined in Additional File 3. The representative themes of the dataset include CBD oil product (topic 1), CBD gummy product (topic 2), perceived efficacy of CBD for migraine/headaches (topic 3), cannabis oil product (topic 4), mixed CBD:THC products (topic 5), hemp oil product (topic 6), transformative experiences with CBD-based products to sleep (topic 7), comparison of products derived from *Cannabis sativa* and *Cannabis indica* (topic 8), adverse events reports of nightmare and vivid dreams (topic 9), CBD legal status (topic 10), mixed CBD:CBN products (topic 11).

### Manual grouping of tweets

The topic analysis highlighted a potential preference within the community for specific types of formulations and concentrations of CBD products. This finding motivated us to further investigate these comments, aiming for a better characterization of preferences and usage patterns of CBD for insomnia within the online community. Due to the magnitude of the signals predicted by the model, manual clustering analysis of tweets into subcategories was conducted solely on the comments that were manually classified as signals (5,408 unique tweets), as outlined in Fig. 2.

**Fig. 5:**
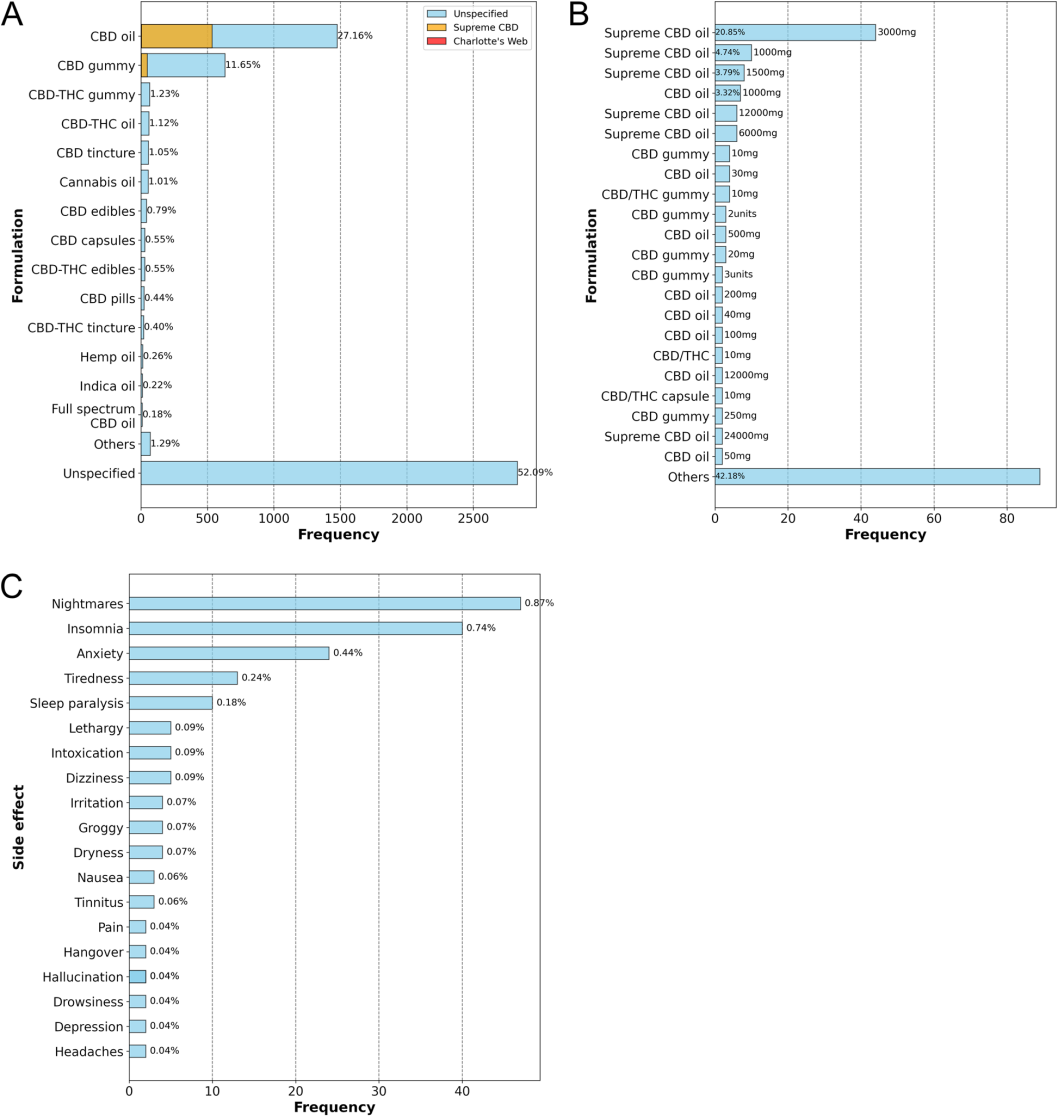
Exploratory analysis based on the manual grouping of *tweets*. **A** Reported CBD preparations in the database. Preparations with fewer than 10 mentions were grouped into the "Other" category, while comments that did not specify the preparation were grouped into the "Unspecified" category. The percentage displayed above the bars indicates the proportion of comments mentioning the preparation relative to the total number of evaluated comments (5,408 *tweets*). **B** Reported CBD concentrations in the database. *Tweets* grouped in the "Others" category corresponded to preparations cited in a single comment. The cited concentration is shown above the respective bars. **C** Adverse events related to the use of CBD for insomnia reported in the database. Adverse events reported in a single comment were grouped into the "Other" category. The percentage displayed indicates the proportion of comments mentioning the event relative to the total number of evaluated comments (5,408 tweets)

The manual grouping of *tweets* in the preparation subcategory (Fig. 5A) revealed a clear preference among the online community for CBD oil and CBD gummies, which together accounted for over one-third of the sample. In contrast, preparations containing CBD combined with other phytocannabinoids were considerably less frequent, being mentioned in fewer than 10% of the tweets.

Interestingly, just under half of the *tweets* provided detailed descriptions of the type of preparation used, and an even smaller proportion included information about the product brand. Only 592 comments referenced brands of CBD products, with a notable mention of the brand SupremeCBD, which was cited in 584 tweets, primarily for oil (536 *tweets*) and gummies (48 *tweets*). Only eight comments mentioned products from the brand Charlotte’s Web, referring to oil (4 *tweets*), gummies (3 *tweets*), and capsules (1 *tweet*).

An even smalller portion of the analyzed tweets—specifically, 211 out of 5,408 tweets—offered additional details about the concentration used or the dosage (Fig. 5B). In these tweets, users' descriptions were often vague regarding the dosage, revealing a common confusion between the product's concentration, the total amount CBD in the product, and the administered dose, as illustrated in Table 3. On the other hand, only three tweets from the database provided precise descriptions of the dosage of CBD used for insomnia (Table 3).

**Table 3:** Examples of *tweets* providing dosage/concentration information for CBD use

Description Preciseness	Representative *Tweets*
*Imprecise*	*• 1000 mg of cbd under my tongue, Feeling weak but can’t sleep Just wish I was numb;*
	*• 5 tips for a successful night of a sleep: 1. Bedroom temp a cool 66°; 2. 15 drops of #CBD oil; 3. Nag Champa incense burning; 4. Breathe Right strip; 5. Brux night guard (may not apply) #sweetdreams*
	*• @twitter_user @twitter_user @twitter_user I'm a terrible sleeper and have been on the 3000 mg for 10 days it has definitely improved my sleep but I'm thinking I could do with trying the 6000 mg im a big bloke so my thinking is the stronger stuff would improve even more any advice?*
	*• @twitter_user @twitter_user @twitter_user @twitter_user @twitter_user @twitter_user @twitter_user 1500 mg I got but. My sleep used to be soo bad it’s u real I sleep like baby without waking every night now*
	*• @twitter_user @twitter_user The 3000 mg oil has worked wonders for my anxiety and sleep*
*Precise*	*• @twitter_user Hey, I write about this for a living and use CBD. Yes, stick with the tincture for bioavailability and onset. I use a 1200 mg oil nightly, 50 mg. I sleep terribly without it. My husband uses 50 mg CBD gummies to sleep, and he works third shift*
	*• @twitter_user @twitter_user Currently nearly at the end of 12000 mg big daddy. Already gone through 2* × *3000 mg before that n only got a few gummies left. Helps massively with sleep n just calms me down abit. Half a dropper in morning, 2 gummies late afternoon n half a dropper before bed. It’s magic*
	*• So I've been on a new CBD regiment for Osteoarthritis and other joint pains!!! Anyone else use CBD oil?? I'm using a 3000 mg, 60 ml bottle. I'm dosing at around 50–60 mg a night for sleep and pain relief. So far, I've been much better with sleep and pain #CBD #cbdwellness #cbdoil*

Forty-four comments referenced the SupremeCBD oil at a concentration of 3000 mg, followed by equivalent products at concentrations of 1000 mg and 1500 mg, mentioned in 10 and 8 comments, respectively. The 1000 mg concentration for CBD oil without a specified brand also appeared in 7 comments. Overall, products with concentrations of ≤ 3000 mg were substantially more frequently mentioned in the database compared to those with higher concentrations (Fig. 5B).

Furthermore, we manually reviewed the database to identify adverse events associated with CBD use (Fig. 5C). A total of 199 adverse events were reported in 169 comments, representing a small fraction of the database analyzed, *i.e.*, 3.12% (169/5,408 comments). Most adverse events were mild to moderate in severity, with only one comment reporting a severe event—atrial fibrillation. Notably, the main adverse events reported in our database include nightmares (0.87%), insomnia (0.74%), anxiety (0.44%), fatigue (0.24%), and sleep paralysis (0.18%). Finally, driven by its high frequency within the database and a CBD concentration that meets medical cannabis regulation (< 0.2% THC) (Souza et al. 2022), we conducted an active search to identify tweets reporting the use of SupremeCBD oil in the signals database (predicted + manually labeled). A total of 3,150 comments with this description were confirmed by specialists and subsequently evaluated in the sentiment analysis.

### Named entity recognition

To complement and systematically extend our previous manual grouping analysis (see Sect. 3.2.2), we employed an LLM-based NER approach to identify key entities within the predicted signal tweets dataset. The predictive performance of the fine-tuned models developed to identify each of the following entities—CBD formulation (subcategory 1), CBD concentration (subcategory 2), and adverse events associated with CBD use (subcategory 3)—are summarized in Table 4.

**Table 4:** Predictive performance of fine-tuned NER models on the test set

NER model	Accuracy	Precision	Recall	F1-score
CBD preparation (subcategory 1)	0.94	0.76	0.71	0.73
CBD concentration (subcategory 2)	0.77	0.71	0.88	0.78
Adverse events (subcategory 3)	0.98	0.76	0.94	0.84

Overall, the NER models effectively identify entities across their respective subcategories (Table 4), demonstrating solid performance with accuracy ranging from 77 to 94% and F1-scores between 0.73 and 0.84. Notably, the model for recognizing adverse events within the tweets achieved the highest performance, demonstrating its superior ability to identify these key entities in the data. This outcome indicates that the performance of the fine-tuned models is suitable for further evaluation of the remaining, non-manually curated database, specifically the predicted signal database (Fig. 2).

**Fig. 6:**
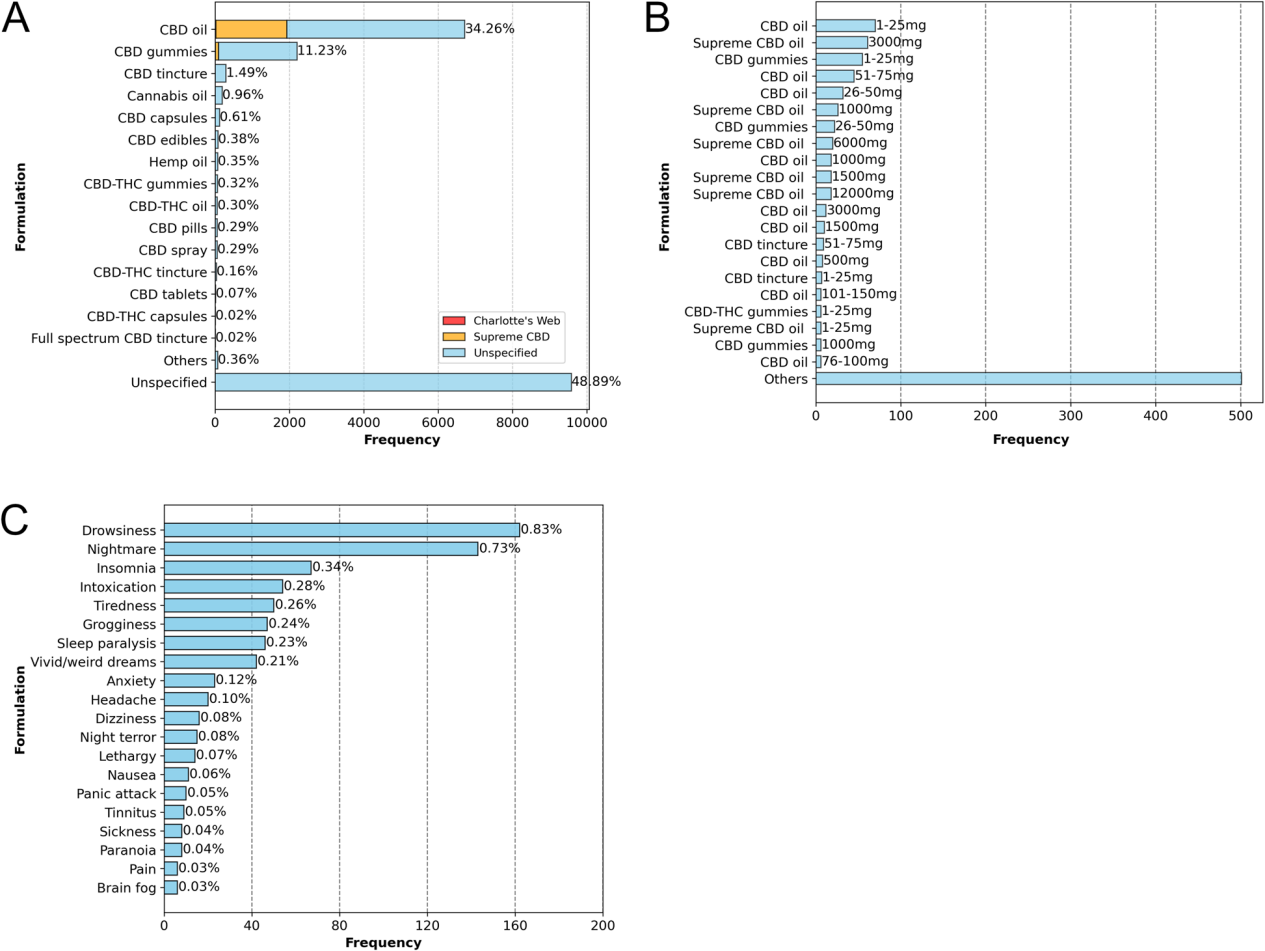
Named entity recognition (NER) analysis within the predicted signal tweets. **A** Predicted CBD preparations in the database. The graph displays the most frequent CBD preparations, with less frequent ones categorized as "Other." Comments that did not specify the preparation are grouped under the "Unspecified" category. The percentage above each bar represents the proportion of comments mentioning the preparation relative to the total number of evaluated tweets (19,597). **B** Most frequent CBD concentrations per product predicted in the database. Tweets categorized as "Others" correspond to less frequent CBD concentrations. The specific concentration is indicated above the respective bars. **C** Most frequent adverse events associated with CBD use for insomnia predicted in the database. Less frequent adverse events mentioned in a single tweet are grouped under the "Other" category. The percentage near each bar shows the proportion of comments mentioning the event relative to the total number of evaluated tweets (19,597)

The results of the NER analysis within the predicted signal database, presented in Fig. 6, reveal that CBD oil was the most frequently used formulation (34.26%), followed by CBD gummies (11.23%). A significant portion of respondents (48.89%) reported using other unspecified CBD products, generically referred to as “CBD”. Analysis of specific brands revealed that SupremeCBD’s oil and gummy products were among the most consumed, highlighting the brand's prominence within the dataset.

Most experiences within the predicted signal database reported a wide variation in the concentrations, total amounts of cannabinoids used, or dosages (Fig. 6), which were often not clearly specified and frequently confused. The data revealed the presence of "Supreme CBD oil," alongside unspecified CBD oil products (with no brand mentioned), across a broad range of milligram strengths, spanning from as low as 1-25 mg to as high as 12,000 mg. Among these, the 1-25 mg and 3000 mg variants emerged as the most popular, reflecting the preferences and usage patterns among CBD consumers.

Regarding side effects (Fig. 6C), a total of 814 side effects were reported in the database. Among these, drowsiness emerged as the most frequently mentioned adverse event, with 162 mentions (0.83%), followed by nightmares, which were mentioned in 143 tweets (0.73%). Other side effects, including insomnia, intoxication, tiredness, grogginess, sleep paralysis, and vivid or unusual (“weird”) dreams, were also frequently mentioned, each appearing in at least 40 tweets. Notably, our NER analysis identified 15 self-reports of night terrors as sleep-related side effects associated with CBD containing products, which were confirmed through manual inspection.

## Analysis of patients’ perception

### Sentiment analysis

Through sentiment analysis, we initially assessed the intensity and polarity of all comments in the database using the compound scores generated by the VADER method for the signals database (group 1). We, then, specifically focused on personal experiences with SupremeCBD oil (group 2).

The sentiment scores were used to assess patient satisfaction with CBD for insomnia based on personal experiences. To guide our selection of the most appropriate statistical tests for subsequent analyses, we applied the Shapiro–Wilk test (Khatun 2021) to the sentiment scores, which can vary continuously from 1 to −1.

The results from the Shapiro–Wilk test indicated that the sentiment score data from both the signals database (group 1) and the SupremeCBD database (group 2) do not conform to a normal distribution (Table 5). For the signals database, the Shapiro–Wilk statistic was 0.91, accompanied by a *p*-value very close to zero, thus, rejecting the null hypothesis of normality. Similarly, the SupremeCBD database exhibited a Shapiro–Wilk statistic of 0.88, with an exceedingly low *p*-value of 5.44 × 10^43^. These findings have important implications for subsequent statistical analyses, highlighting the necessity for non-parametric methods (Khatun 2021).

**Table 5:** Results of the hypothesis tests conducted

Statistical Test	**Signals Database (Group1)**		**SupremeCBD (Group2)**	
	*statistic*	*p-value*	*statistic*	*p-value*
Shapiro–Wilk	.91	.00***	.88	5.44 × 10^–43^***
Wilcoxon Signed-Rank^&^	2.2 × 10^08^	.00***	3.9 × 10^06^	4.12 × 10^–234^***
Phillips-Perron	−5.32	4.93 × 10^–06^ ***		
Augmented Dickey–Fuller	−4.38	3.15 × 10 − 4***		

^***^ *p-value* < 0.001. *&*: test conducted comparing the group with a theoretical distribution with a median equal to zero, considering the one-tailed alternative hypothesis in the “greater than” direction

Nevertheless, the Shapiro–Wilk test can exhibit sensitivity to deviations from normality in large samples (*n* > 5,000), where even minor deviations may result in significant rejections of the null hypothesis (Boedec 2019). Given that our signals database (group 1) comprises *n* = 25,005, additional analyses are warranted to confirm these findings, including a visual inspection of the distribution.

Therefore, we plotted a Kernel density graph for the sentiment score values from the signals database. This is a non-parametric approach used to estimate the function of unknown probability densities (Zhong et al. 2024). The Kernel density graph overcomes some limitations of traditional distribution representations, such as histograms, which are highly dependent on the number and interval of the bars. Thus, the proposed representation favors the identification of trends in the data, such as shape, peaks, multiple modes, or asymmetries, providing a better understanding of the evaluated distribution (Ghaderi et al. 2022). This analysis indicated a distribution that is very far from Gaussian, corroborating the results of the Shapiro–Wilk test (Fig. 6A).

**Fig. 7:**
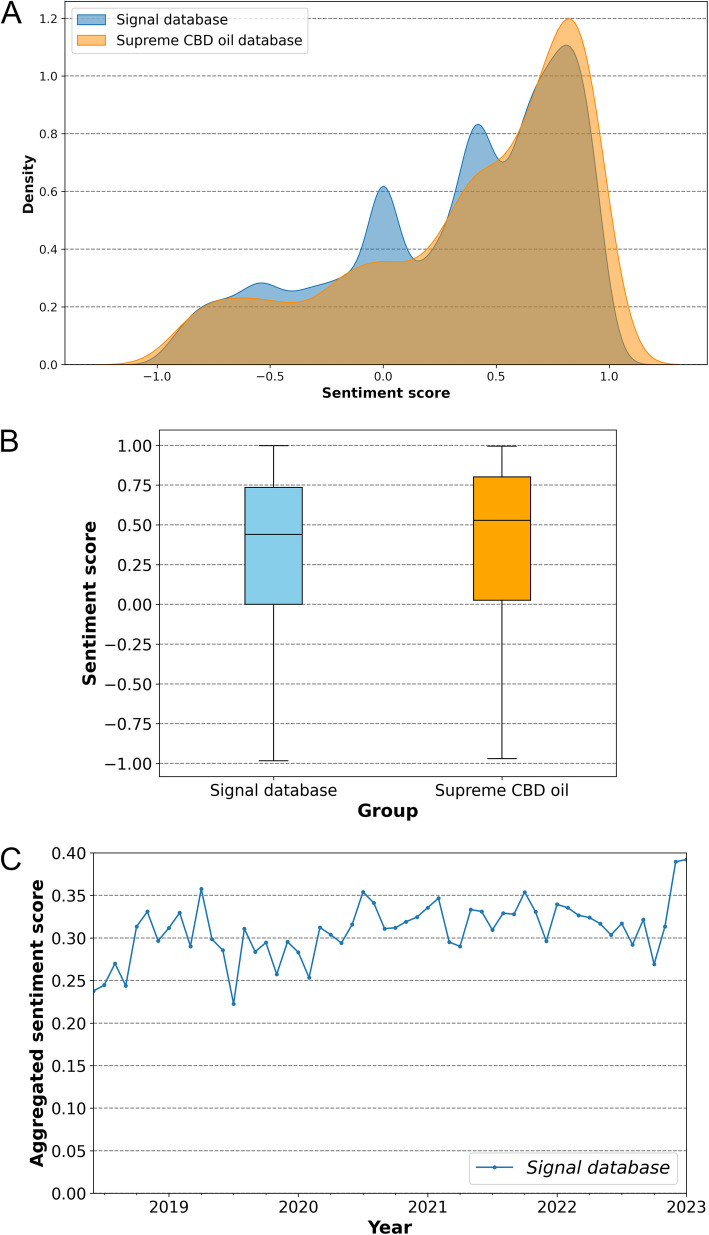
Sentiment analysis using the VADER method of Twitter comments on personal experiences with CBD for insomnia. **A** Kernel density plot illustrating the distribution of sentiment scores (compound scores). The blue curve represents the general sentiment trends across the broader signal database containing experiences with CBD-containing products, while the orange curve represents sentiment trends specifically related to experiences with Supreme CBD oil. **B** Box plot showing the distribution of sentiment score computed from the signal database (blue) and the Supreme CBD Oil database (orange). The median of the distribution is represented by a black line. **C** Aggregated monthly average sentiment score values derived from user experiences in the signal database, which contains feedback on CBD-containing products spanning from the FDA approval of Epidiolex on June 25, 2018, up to January 9, 2023. These values capture the evolving consumer sentiment towards CBD products over this period, providing a view of public perception and potential shifts in sentiment following this major regulatory milestone

The kernel distribution also reveals a clear inclination towards positive experiences compared to negative and neutral ones. This trend is also evident in the box plot representation (Fig. 7B). The median polarity scores for comments in the signals database and SupremeCBD were 0.44 and 0.52, respectively. This indicates that the first two quartiles—representing 50% of the sample—correspond to positive experiences, with sentiment scores ≥ 0.44, significantly greater than zero. Additionally, the lower bound of the third quartile (Q3) is equal to or greater than zero for the signals database, while for the SupremeCBD database, it is greater than zero. Therefore, at least 75% of experiences in the first sample were neutral or positive, while more than 75% of experiences in the second sample were positive. These results reinforce the positive trend of reported experiences, particularly in the SupremeCBD database.

After confirming the non-normality of the groups and identifying a positive polarity trend in the database comments, we employed the non-parametric Wilcoxon signed-rank test to compare the median of each group against a hypothetical distribution with a median of zero (Rosner et al. 2006). The alternative hypothesis was that the median of the signals database is statistically greater than zero (alternative = “greater than”). The test statistics (Z) for the signals and SupremeCBD databases were 2.2 × 10^8^ and 3.9 × 10^6^, respectively, with extremely low *p*-values approaching zero (Table 1). These results provide statistical evidence at the 0.05 significance level to reject the null hypothesis, indicating that the medians of the evaluated groups are statistically greater than zero. This suggests predominantly positive feedback regarding the use of CBD for insomnia. Therefore, our findings propose that CBD could be beneficial for this clinical condition.Finally, we conducted a time series analysis (Fig. 7C) to investigate whether perceptions regarding the off-label use of CBD for insomnia were influenced by the FDA's approval of Epidiolex on June 25, 2018 (Huestis et al. 2019). In this analysis, we did not assess sentiment polarity over time for the SupremeCBD database (group 2) since the company registered its trademark on December 18, 2019 (Registration Nº: 12,370,423) and began marketing its products only after that date. As a result, it was not possible to evaluate the impact of Epidiolex's approval on this group, as the brand had not yet been established in the market during this key milestone.

The monthly average sentiment scores from *tweets* in the signals database (group 1) were presented as a time series, enabling the evaluation of opinion polarity following the approval of Epidiolex (Fig. 7C). A visual inspection of the time series reveals stationary behavior, marked by stable values over time with minor fluctuations around a constant mean (Silva et al. 2021). To confirm the stationarity of the series, we initially conducted the Phillips-Perron statistical test, which assesses the null hypothesis that the time series can be modeled by a unit root with a time-dependent structure. The test statistic (PP) was −5.32, resulting in a *p*-value of 4.93 × 10^–6^ (Table 1), which is significantly lower than 0.05, thus rejecting the null hypothesis and confirming its stationarity (Wong and Nguyen 2022). This finding reflects the absence of a trend or seasonality in the data, indicating that the computed values are not dependent on time (Da’Ar OB, Ahmed AE. Underlying trend, seasonality, prediction 1878). Consequently, it suggests that patients’ perceptions of CBD use for insomnia may not have been affected by the FDA's approval of Epidiolex®.

The Augmented Dickey-Fuller test was then performed to further investigate the stationarity of the sentiment series. The analysis resulted in a test statistic of −4.38 with a *p*-value of 3.15 × 10⁻4, which is well below the 0.05 threshold, providing strong evidence to reject the null hypothesis of non-stationarity. As a result, the data do not show a significant trend or seasonality over time, and the observed fluctuations in polarity are random, rather than indicative of systematic changes (Silva et al. 2021), such as those associated with the approval of Epidiolex®.

## Discussion

Our primary objective was to investigate patients' perspectives on the off-label use of CBD for insomnia through sentiment analysis, providing insights to enhance clinical decision-making and cost-efficiency (Wankhade et al. 2022). We then compiled and processed a database of 74,562 tweets by utilizing an extensive list of keywords related to cannabidiol, sleep, and insomnia, as outlined in Fig. 1. This dataset spans from June 25, 2018—the date of Epidiolex's FDA approval (8)—to January 9, 2023. Most tweets in the database were considered “noise”, primarily consisting of advertisements and commercial content (Table 1), and did not accurately reflect user self-reported experiences with CBD in various formulations for insomnia (which we named "signal" tweets).

To resolve this, 7.418 randomly selected tweets were manually labeled, preparing the dataset for fine-tuning a RoBERTa classifier to identify relevant content. An initial round of training did not yield satisfactory performance, likely due to the imbalanced nature of the database, with a signal-to-noise ratio of 28:72. As a result, a second round of manual tweet classification was performed, focusing on the tweets predicted as signal by the initial model. This strategy, illustrated in Fig. 1, allowed us to obtain a more balanced dataset (signal-to-noise ratio of 48:52) for training a refined model, which demonstrated substantially improved performance on the test set (Fig. 3).

In this study, which focused on screening *tweets* with a higher likelihood of being signals for further analysis by specialists, it was essential to ensure the reliability of predictions for the positive class (“signal”) to avoid unnecessary time expenditures (Ng and Henikoff 2003). Consequently, the refined RoBERTa model, which achieved high performance metrics for the positive class—recall (0.94), precision (0.86), and F1-score (0.9), all approaching 1—was deemed suitable for classifying the remaining unseen tweets in the database.

After constructing the signal database, which contained only relevant tweets reflecting self-reported experiences with CBD products for insomnia, we preprocessed the tweet text to enable topic modeling and identify potentially significant themes within the dataset, as outlined in Fig. 2. Topic modeling involves a set of algorithms used to identify hidden topics in unstructured datasets, proving to be an efficient tool for handling large volumes of data, such as our signal dataset. Through the automation provided by topic modeling, data is organized and simplified to facilitate the understanding of the underlying context, offering insights into the main themes and concepts addressed (Qomariyah et al. 2019).

This analysis revealed that the optimal number of topics (k) for maximizing the coherence score—an indicator of interpretability and the quality of the topics identified by the model (Min et al. 2020)—was 11, when using bigrams instead of unigrams (Fig. 4A). The topic analysis identified recurring themes related to the perceived effectiveness of CBD for various health conditions, along with practical considerations such as concentration, treatment routines, and preferences for specific preparations (Table 2).

Furthermore, we integrated the BERTopic algorithm, based on large language models (LLMs), to enhance and complement our topic analysis. While LDA remains a well-established method for topic modeling, state-of-the-art approaches like BERTopic offer superior ability to capture contextual semantics and the grammatical roles of words, providing deeper insights into the underlying themes within the data, as demonstrated by Uncovska et al. (2023) (Uncovska et al. 2023).

BERTopic revealed new topics (Additional File 3) that were either less prominent or not fully explored by LDA in our dataset (Table 2). For instance, "Mixed CBD:THC products" (Topic 5) and "Mixed CBD:CBN products" (Topic 11) were identified as distinct themes, reflecting the growing interest in hybrid cannabinoid formulations. Additionally, a representative subset of tweets explicitly compared products derived from *Cannabis sativa* and *Cannabis indica* in treating insomnia and sleep disorders (Topic 8), providing valuable insights into patients' perspectives on the specific benefits of each strain. The legal status of cannabis, a prevalent topic within the dataset, was also revealed by BERTopic in "Topic 10". Additionally, BERTopic's inclusion of "Adverse events reports of nightmares and vivid dreams" (Topic 9) emphasizes its ability to detect more complex and evolving trends.

While LDA also captured product-specific experiences, BERTopic provided a clearer and more detailed picture of how users interact with different CBD preparations. Topics related to hemp oil (Topic 6) and cannabis oil (Topic 4) emerged more distinctly, offering a finer breakdown of CBD-based product preferences. These granular topics enriched the discussion by highlighting varying user experiences with different types of CBD oil—insights that LDA would likely have collapsed into broader categories. Ultimately, both LDA and BERTopic highlighted the perceived effectiveness of CBD in treating various health conditions, such as anxiety, pain (Table 2), headaches, and migraines (Additional File 3), as well as user satisfaction with its effects to insomnia, particularly in the context of "Transformative experiences with CBD-based products for sleep" (Topic 7).

Our topic modeling analysis prompted a deeper investigation into users' preferences and usage patterns of CBD for insomnia within the online community. Given the large volume of predicted signals, a manual grouping analysis was conducted on the 5,408 tweets manually labeled as signals in the database to explore the subcategories of "CBD preparation," "CBD concentration", and "adverse events" in greater detail, as outlined in Fig. 2.

The manual assessment of the "CBD preparation" subcategory revealed a clear preference for CBD oil and CBD gummies over preparations containing other phytocannabinoids (Fig. 5). Most tweets did not specify the type of preparation used, with only around 10% mentioning a product brand. Among these, SupremeCBD oil stood out due to its notable frequency in the database. This suggests that users tend to focus less on specific product details when sharing their experiences online. The analysis of the "CBD concentration" subcategory revealed a significant gap in how users report dosage on social media, with many confusing it with product concentration or the total CBD content in the product. This discrepancy renders our database unsuitable for studying CBD dosage in the context of insomnia. Nevertheless, it was possible to identify the main CBD preparations (Fig. 5B), with the majority consisting of products containing concentrations ≤ 3000 mg, rather than those with higher concentrations (Fig. 5B). This may be related to the high cost of CBD products available on the market, which tend to become even more expensive at higher concentrations, making them less accessible (Smart et al. 2017; Amann et al. 2022).

After analyzing the "CBD preparation" and "CBD concentration" subcategories, which primarily reflect patients' preferences regarding CBD products, we proceeded with a manual assessment of the self-reported "adverse events" subcategory. Studies have shown that adverse events caused by medications after their introduction to the market represent a significant public health issue, accounting for up to 5% of hospital admissions, 28% of emergency visits, and 5% of hospital deaths. Therefore, post-marketing pharmacovigilance is crucial for the pharmaceutical industry and regulatory agencies (Xu and Wang 2014).

Although it is mandatory for manufacturers to report adverse events related to their medications, reporting by healthcare professionals and users is voluntary, posing a significant challenge for pharmacovigilance. Due to the limitations of spontaneous reporting systems—such as restricted access to electronic health records and medical charts, which are typically only available to affiliated physicians and healthcare centers—researchers have been encouraged to explore additional data sources. In this context, the rise of social media and the availability of natural language processing tools have opened new opportunities for pharmacovigilance. This is particularly relevant, as reports of adverse events originating from social media are subject to official review by regulatory agencies like the FDA (Sarker et al. 2015). Furthermore, monitoring adverse events on social media offers several operational advantages, such as the extensive availability of up-to-date, freely accessible data generated spontaneously by patients themselves (Harpaz et al. 2014).

Therefore, the monitoring of adverse events proposed in this study holds significant importance, as it provides a comprehensive understanding of the potential risks associated with the off-label use of CBD for insomnia, thereby complementing existing safety data. Identifying and documenting these events not only yield crucial insights into the safety and tolerability of CBD but also facilitate a holistic evaluation of its benefit-risk profile. By incorporating adverse events into sentiment analysis, we can enhance our overall understanding of CBD usage and support informed clinical and regulatory decisions regarding this substance.

The analysis of adverse events identified a total of 199 occurrences reported across 169 comments, with the majority being mild to moderate in severity (Fig. 5C). Clinical trials have indicated that cannabidiol is quite safe, showing an incidence rate of 9.7% for adverse events in patients with epilepsy, and only 1.2% for severe adverse events (Fazlollahi et al. 2023). Thus, our findings point to a relatively lower rate of adverse events (3.12%) compared to the rate reported in the literature (9.7%), which is expected, as reporting of these events is mandatory in clinical trials and spontaneous in our database (Sarker et al. 2015).

In our database, most self-reported adverse events linked to CBD usage were mild to moderate in severity, with a single comment noting a severe event—atrial fibrillation. Although atrial fibrillation was not directly associated with CBD use (U.S. Food and Drug Administration 2018), the study by Patel et al. (2021) demonstrated that individuals with cannabis use disorder had up to a 50% higher risk of developing cardiac arrhythmias, including atrial fibrillation. Despite their limited occurrence (< 1%), the main adverse events reported in our database included nightmares, insomnia, anxiety, fatigue, and sleep paralysis. While cannabidiol is recognized for its anxiolytic effects (Babson et al. 2017), it is important to note that it can also cause insomnia and agitation as adverse events, albeit less frequently (Abu-Sawwa et al. 2020). Furthermore, the effect of CBD on modulating REM sleep latency has been demonstrated in pre-clinical rat models, although it has yet to be confirmed in humans (Hsiao et al. 2012). This may be related to occurrence of nightmares and sleep paralysis reported by the Twitter users in our database, as these phenomena are REM-dependent (Denis 2018).

Another potential factor contributing to the occurrence of these adverse events is the presence of mixed CBD preparations that contain THC, which is recognized for its predominantly anxiogenic effects (Sharpe et al. 2020). Nonetheless, as previously discussed, the frequency of mixed preparations in the sample was considerably lower than that of pure CBD preparations (Fig. 5A). Therefore, it is more likely that the profile of adverse events reported is primarily attributable to CBD.

As reviewed by Lavender et al. (2024), the adverse effects of CBD and other cannabinoids for sleep disorders, including insomnia, vary depending on dosage, duration, composition, and study reporting methods. Smaller studies with more intensive measures tend to report more adverse events, while larger studies often lack standardized adverse events collection protocols, highlighting the need for consistency. Overall, most adverse events are mild and self-limiting. Only two out of seven studies reported severe adverse events, including restlessness, tachycardia, and dizziness, which were resolved through dosage reduction (Lavender et al. 2024).

Among clinical trials focusing on pure CBD preparations for insomnia, Narayan et al. (2024) found that dry mouth was the most common side effect (51.67% of overall occurrences), occurring significantly more frequently in the CBD group compared to the placebo. Other mild side effects, such as nausea, light-headedness, diarrhea, dizziness, anxiety, and paranoia were also more prominent in the CBD intervention group, though no significant differences were observed compared to the placebo (Narayan et al. 2024). In the open-label, multi-arm trial conducted by Saleska et al. (2022) to evaluate the safety and effectiveness of commercially available CBD-predominant products for various conditions, including sleep disturbance. Mild to moderate adverse events were reported, including gastrointestinal upset (1.2%), headaches (1.4%), mental fog (1.1%), chest pain (1.1%), nausea (1.1%), trouble falling asleep or staying asleep (0.1%), anxiety (0.1%), irritability (< 0.1%), depressed mood (< 0.1%), fatigue (< 0.1%), and dry mouth (< 0.1%) (Saleska et al. 2022). Thus, the adverse event profile reported in our database (Fig. 5) is consistent with findings from clinical trials.

Leveraging the high frequency of SupremeCBD oil mentions, as revealed by the manual grouping analysis (Fig. 5A), we conducted an active search in our signals database (predicted + manually labeled) that yielded a substantial number of tweets—3,150 in total—reporting the use of this specific product. SupremeCBD oil contains less than 0.2% THC, which enables a more precise investigation of CBD’s effects, given that the low THC concentration significantly mitigates the risks of unwanted psychoactive effects and reduces the pharmacological impact associated with THC (Casati et al. 2022). Consequently, this facilitates a more targeted evaluation of the therapeutic benefits of CBD in treating insomnia, with this subset (group 2) being independently analyzed during the sentiment analysis, conducted in parallel with the entire signals database (group 1).

To analyze the entire signal database (predicted + manually labeled), comprising 25,005 tweets (Fig. 1), we successfully fine-tuned individual NER models, which demonstrated robust performance (Table 4) in detecting key entities within the database. These models were specifically applied to the previously unexplored predicted signal subset of 19,597 tweets, enabling the efficient extraction of relevant entities from three key subcategories: CBD formulation (subcategory 1), CBD concentration (subcategory 2), and adverse events (subcategory 3).

Overall, this outcome, presented in Fig. 6, revealed a pattern consistent with the findings previously reported and properly discussed for the manually labeled database of 5,408 tweets, reflecting the community's general preferences and self-reported adverse event profile of CBD products (Fig. 5). This consistency highlights the reliability of the fine-tuned NER models, demonstrating their efficiency in processing large datasets while preserving the accuracy of extracted entities, owing to their ability to effectively capture context (Perera et al. 2020). Additionally, this analysis uncovered night terrors and vivid dreams as sleep-related side effects associated with CBD-containing products, demonstrating the potential of automated NER tools to identify less commonly reported or overlooked side effects, thus enriching the overall understanding of CBD's safety profile.

Patient perception, or “*feedback*”, is a term used to describe a patient's opinion, satisfaction, or viewpoint regarding the quality of a particular treatment or healthcare service. Although there are various strategies to capture patient feedback, its use is generally limited to hospital administration and management contexts. With the increased access to the internet and the popularization of social media, feedback has become a social activity, where timely and unsolicited comments authentically express patients' perceptions. In this context, sentiment analysis proves effective in understanding the intensity and polarity of opinions, providing relevant information to assess satisfaction and perceived efficacy regarding a specific treatment or intervention (Pandey et al. 2023).

Our analysis revealed that the sentiment scores for both groups of tweets, *i.e.*, entire signals database (group 1) and SupremeCBD tweets (group 2), displayed a non-normal distribution (*p* < 0.01) (Table 5) and an overall positive polarity trend (Fig. 7). We also verified through statistical hypothesis that the median sentiment scores for both groups were significantly greater than zero (*p* < 0.01) (Table 5), reinforcing the predominantly positive feedback regarding the use of CBD for insomnia. These results suggest that CBD may indeed offer therapeutic benefits for managing insomnia, as reflected by the general sentiment expressed in the analyzed tweets.

Studies in animal models have investigated the effects of CBD on sleep quality and the sleep–wake cycle. Chagas et al. (2013) reported an increase in total sleep time in rats following medium and high doses of CBD compared to a placebo. The study also indicated that medium doses of CBD were able to reduce REM sleep latency. Similarly, Hsiao et al. (2012) observed that CBD blocked REM sleep suppression induced by anxiety, thereby improving sleep quality. This finding is supported by a case report in which CBD administration reduced the occurrence of insomnia symptoms and sleep disturbances related to post-traumatic stress disorder. These results suggest that CBD's anxiolytic effects may contribute to improved sleep quality (Babson et al. 2017).

A crossover study involving individuals with insomnia suggested that CBD doses of 160 mg/day were able to increase total sleep time and reduce the frequency of nighttime awakenings (Babson et al. 2017). Shannon et al. (2019) investigated the effects of CBD on sleep through a case series involving 72 individuals, who received doses of up to 175 mg/day for three months. The study showed a significant improvement in the Pittsburgh Sleep Quality Index in 66.7% of patients. Nonetheless, the benefit was not sustained throughout the three months analyzed. Notably, the open-label and non-randomized nature of this study necessitates cautious interpretation of the findings, requiring further randomized controlled trials to provide definitive clinical guidance (Shannon et al. 2019).

An open-label, multi-arm trial conducted by Saleska et al. (2022) assessed the safety and effectiveness of commercially available CBD-dominant products for various conditions, including sleep disturbances, with a total of 2,816 participants. Participants who were assigned to take a CBD product reported significant improvements in self-reported well-being, anxiety, sleep disturbances, and pain compared to the waitlist control group. Notably, the study has several limitations, including its open-label design, short duration, reliance on self-reported data, and limited generalizability due to the predominantly female and white sample. The study also involved a range of CBD products and did not account for potential confounding factors (Saleska et al. 2022).

Recently, a randomized controlled pilot trial conducted by Narayan et al. (2024) evaluated the efficacy of 150 mg of cannabidiol (CBD) compared to placebo as a treatment for primary insomnia. Participants in the CBD group consistently reported higher well-being scores throughout the trial and demonstrated superior objective sleep efficiency compared to the placebo group. Nonetheless, the study found no significant improvements in key sleep outcomes for the CBD group compared to baseline. Additional controlled trials examining varying treatment periods and doses are necessary to validate and expand upon these results (Narayan et al. 2024).

Currently, several ongoing studies are investigating the use of oral cannabidiol (CBD) for insomnia, with clinical trials registered under the identifiers NCT05840822 and ACTRN12623000803695. In parallel, additional studies are exploring CBD's potential for managing mild sleep disturbances, with trials registered under the identifiers ACTRN12622000464763 and ACTRN12621000632897 (Lavender et al. 2024).

Comprehensive systematic reviews have also been undertaken to investigate the potential of cannabidiol (CBD) in managing insomnia. Among these, Ranum et al. (2023) conducted an extensive review encompassing 34 studies, which indicated that CBD, either alone or in combination with equal quantities of THC, may be effective in alleviating insomnia symptoms. Notably, among studies focused on CBD-predominant treatments, 4 out of 7 reported significant improvements in insomnia outcomes. Nevertheless, only two studies specifically targeted patients diagnosed with insomnia, and many relied on non-validated subjective measures rather than objective assessments (Ranum et al. xxxx). An even more recent review on the use of CBD for various sleep disorders beyond insomnia, published in 2024 by Lavender et al*.*, highlighted a significant rise in the adoption of this intervention within clinical populations. While some isolated findings from limited trials have shown potential benefits, these studies are often constrained by small sample sizes, short durations, and methodological shortcomings (Lavender et al. 2024).

Therefore, the current evidence in the literature is still not considered sufficient to support the efficacy of CBD for insomnia (Lavender et al. 2024; Ranum et al. 2023). In this context, our findings may provide an additional layer of understanding regarding the perceived effectiveness of CBD by users, complementing the results already available. While traditional clinical trials offer only objective data on CBD's efficacy, sentiment analysis provides a subjective perspective by capturing individual user experiences (Denecke and Deng 2015). Therefore, the sentiment analysis presented in this study could contribute to a more comprehensive perspective on CBD's impact on insomnia management, particularly considering the significant gaps in established scientific knowledge.

Finally, we aggregated the data by month to track patients' perceptions of the off-label use of CBD for insomnia over time (Fig. 7), covering the period from June 25, 2018—the approval date of Epidiolex (8)—to January 9, 2023. The approval of the first medication containing a purified active ingredient from *Cannabis spp.*—CBD—may have had a significant impact on public perception of CBD use, potentially influencing the polarity of opinions on the matter (Jabalameli et al. 2022). SupremeCBD products became commercially available only in 2020, more than a year after the approval of Epidiolex. As a result, it was not possible to conduct this analysis for this specific subset (group 2); hence, the analysis was limited to group 1. This analysis indicates that the sentiment score series exhibits stationary behavior over time (*p* < 0.01). Consequently, the data do not exhibit significant trend or seasonality over time, and the observed fluctuations are random rather than indicative of a systematic change. Therefore, the fluctuations observed in Fig. 7 are random variations rather than a response to the approval of Epidiolex® (Wong and Nguyen xxxx; Da’Ar OB, Ahmed AE. Underlying trend, seasonality, prediction 1878).

Thus, public opinions and perceptions expressed on social media regarding the use of CBD for insomnia remained consistent throughout the analyzed period, regardless of significant external events, such as the introduction of Epidiolex® to the market. In this context, the insights derived from the sentiment analysis provide greater confidence and credibility, demonstrating resilience to short- to medium-term fluctuations caused by external events. This strengthens the validity of the drawn conclusions, allowing for a more robust interpretation of patients' perceptions regarding CBD treatment for insomnia.

## Limitations

This study relies on publicly available social media data, which inherently entails certain limitations and raises important ethical considerations. This is especially relevant in the case of emotion-related data—such as that used in this study—which can be particularly sensitive. Despite that, the General Data Protection Regulation (GDPR) does not explicitly classify it as special category data, resulting in limited protections and a lack of well-defined guidelines for its safeguarding (Häuselmann et al. 2023). In response to these ethical concerns, we implemented techniques to mitigate potential risks associated with data processing and availability, including anonymization, and the removal of links or user mentions that could potentially lead to the identification of the authors. Such an approach aligns with current best practices in privacy preservation, significantly reducing the risk of re-identification (Gadotti et al. 2024).

Furthermore, while social media data offers substantial advantages for sentiment analysis—such as access to a large and diverse audience, along with the spontaneous and often candid nature of posts—there are potential drawbacks. These mainly include demographic biases associated with specific platforms. On Twitter, for instance, users tend to be younger, which may not represent the opinions of the overall population. This age skew, along with other factors such as regional and socio-economic differences, poses challenges to the generalizability of our findings (Yang et al. 2023).

## Conclusions

In this study, we compiled a database containing 74,562 unique comments through text mining of *tweets* published between 2018 and 2023, focusing on keywords related to CBD in various preparations and insomnia. Using this database, we trained and validated a ROBERTA-based deep learning model to detect relevant *tweets*—those that discussed personal experiences with off-label CBD use for insomnia. The performance metrics calculated for the model on the test set demonstrate its robustness, effectively identifying predictions within the signal class and achieving a commendable balance between precision and recall.

Topic modeling revealed eleven central themes within the signal tweets database, covering perceived efficacy of CBD for anxiety, pain, and insomnia, as well as practical considerations regarding treatment routines and preparation preferences. Additionally, we manually investigated the main adverse events reported in the database, which indicated a relatively low frequency of events (3.12%), notably including nightmares, insomnia, anxiety, fatigue, and sleep paralysis. Although these adverse events were considerably less frequent compared to drowsiness and other anxiolytic effects, they could be attributed to the dose-dependent biphasic effect of CBD.

Finally, we evaluated patient perceptions regarding off-label CBD use for insomnia. Our findings reveal predominantly positive feedback on reported personal experiences, suggesting a favorable perception of CBD's efficacy for insomnia. Additionally, this study provides an additional layer of understanding regarding the perceived efficacy by users, which aligns with preliminary studies available in the literature. Therefore, it may contribute to a more comprehensive perspective of CBD's impact on insomnia management, especially considering that existing evidence in the literature remains insufficient to determine its efficacy.

## Supplementary Information

Additional file 1. Model architecture and training parameters.

Additional file 2. Tweet database.

Additional file 3. Topic modeling analysis using BERTopic.

## Data Availability

No datasets were generated or analysed during the current study.

## Abbreviations

CBD: Cannabidiol
THC: Tetrahydrocannabinol
FDA: Food and Drug Administration
REM: Rapid Eye Movement
POS: Part of Speech
LDA: Latent Dirichlet Allocation
NLP: Natural Language Processing
TF-IDF: Term Frequency-Inverse Document Frequency
VADER: Valence Aware Dictionary and sEntiment Reasoner
ROBERTA: Robustly Optimized BERT Pretraining Approach
