# Supplementary material for: Assessing patient perceptions of off-label cannabidiol use for insomnia through sentiment analysis
Source: J Cannabis Res. 2025 Nov 19;7:94. doi: 10.1186/s42238-025-00306-7 (PMC12628953; doi:10.1186/s42238-025-00306-7)
Supplement: Supplementary file 1 — Additional file 1. Model architecture and training parameters. [file 42238_2025_306_MOESM1_ESM.pdf]

Additional File 1. Model Architecture and Training Parameters.

The model architecture comprised three layers: an input layer with 768 densely connected input units (neurons), a hidden layer with 32 densely connected neurons, and an output layer with a single neuron. This architecture was designed to facilitate binary classification, where the output represents the probability of a given class, specifically signal tweets. The ReLU activation function was applied to the inputs of the first layer, introducing non-linearity to the network and enabling it to learn more complex relationships within the data. Finally, the Sigmoid activation function was used after the fully connected second layer to convert the output into a probability between 0 and 1, indicating the likelihood of the tweet belonging to the signal class.

The following hyperparameters were used for training the model: padding = “max_length”, max_length = 150, truncation = True, return_tensors = “pt”, loss function = BCELoss, optimizer = Adam, number of training epochs = 30. The model's loss and accuracy on the training and validation sets were computed at each epoch. Early stopping was implemented to halt training if there was a significant decrease in accuracy or an increase in loss for the validation set compared to the previous epoch.
