# Supplementary material for: Assessing patient perceptions of off-label cannabidiol use for insomnia through sentiment analysis
Source: J Cannabis Res. 2025 Nov 19;7:94. doi: 10.1186/s42238-025-00306-7 (PMC12628953; doi:10.1186/s42238-025-00306-7)
Supplement: Supplementary file 3 — Additional file 3. Topic modeling analysis using BERTopic. [file 42238_2025_306_MOESM3_ESM.pdf]

Additional file 3. Topic modeling analysis using BERTopic.

Topic	*tweets*	Name	Representative bigrams	Representative Tweets
**1**	10034	CBD oil	*sleep cbd, tried cbd, take cbd, use cbd, taking cbd, cbd oil*	• @twitter_user I use a Cbd oil to help with sleep and it works fine<br>• @twitter_user CBD oil’s helped me with anxiety and sleep!<br>• @twitter_user I have severe insomnia and it helps sometimes. What really works for me is CBD oil.
**2**	3062	CBD gummy	*cbd sleep, cbd gummies, sleep gummies, take cbd, tried cbd*	• so thankful for cbd gummies to help me sleep 👌<br>• @twitter_user I use CBD gummies to help me sleep, and they work great.<br>• @twitter_user @twitter_user @twitter_user I gave gummies a try as I struggle to get quality sleep,,,,,,Best sleep I’ve had in years !!
**3**	184	Perceived efficacy of CBD for migraine/headaches	*migraine insomnia, chronic migraine, have migraines, have migraine, from migraines, cbd products*	• 8:1 CBD is by far the best thing for my migraines & helping me sleep/relax.<br>• @twitter_user See CBD oil helps my migraines a shit ton and my insomnia. And I already have IBS so yknow what it’s all good.<br>• @AletheiaAtheos I’ve found cbd oil to be BiG help when I have migraine, it puts me to sleep, so maybe that would help.
**4**	139	Cannabis oil	*oil sleep, cannabis oil, take cannabis, use cannabis, tried cannabis, best sleep*	• I took cannabis oil to help me sleep but I’m so high 🥴<br>• @twitter_user @twitter_user @twitter_user Agree.. since I use cannabis oil I sleep like a baby . But I have a feeling @twitter_user doesn’t favorisiert cannabis oil?? Am I wrong ? 🤔<br>• @twitter_user I take cannabis oil for sleep. It wasn't enough. 🙄
**5**	123	CBD with THC products	*take cbd, use cbd, cbd oil, some cbd, havent smoked, never smoked*	• Nope. I use cbd+ and thc+ based products for anxiety, insomnia, endo symptoms etc. but no smoking. Just ingesting via liquid or applying topically, usually. https://external_URL',<br>• @twitter_user I take CBD/THC capsules for sleep but I dont smoke it. It's been a long time<br>• @twitter_user @twitter_user Never smoked, but use edibles for sleep and ease of inflammation. Works. Look into, start with small doses of CBD/THC
**6**	116	Hemp oil	*hemp oil, cbd hemp, take hemp, hemp cbd, try hemp, hemp products*	• '@twitter_user it’s an oil that comes from the hemp plant and it helps with anxiety and insomnia.... cbd and hemp oil are actually really good for you, I’ve been using hemp oil at night for about two weeks and have been sleeping better and having less

				digestion issues and bloating<br>• @twitter_user They work. Plain ole hemp oil is much cheaper and it really works for sleep and pain. I like CBD better but the cost is too much for me but the hemp oil works pretty darn good.<br>• My bfs mom bought us CBD oil and hemp oil and I took some hemp oil to help me sleep and wow am I fading fast
**7**	107	Transformative experiences with CBD-based products to sleep	*cbd oil, recommend cbd, cbd spray, cbd tincture, sleep like, tongue helps*	• @twitter_user @twitter_user Cbd spray under the tongue has been wonderful for my sleep and muscle pain it’s a tad expensive but I’d say it’s definitely worth it<br>• @twitter_user Try CBD oil! Works wonders on me, 6% three drops under the tongue and I sleep like a baby<br>• @twitter_user I'd straight up just take some CBD tbh. I currently have a small spray, just one spray under the tongue every night just before bed and I sleep like I'm in a coma. Been doing that for three years now, absolute life changer.
**8**	106	Comparison of Products Derived from *Cannabis sativa* and *Cannabis indica*	*sleep sativa, indica cbd, cbd indica, indica works, like sativa, sativa makes*	• @twitter_user Two main strains are Indica and Sativa. Really the strain not matter with topicals, IMHO. I use Indica that I make into an oil to subdue muscle spasms incurred by a traumatic brain injury. Indica good for sleep. Sativa makes my heart race, no like.<br>• @twitter_user Is this to help you sleep? If not don't be surprised if it makes you hella sleepy. YMMV but generally, indica=drowsy satvia=stimulation (or if you have ADHD = less drowsy). My CBD oil is a Satvia and it still puts me to sleep when I take it.<br>• @twitter_user @twitter_user Indica or Indica with CBD. For great relaxation and sleep, body high. Sativa makes me too anxious, it is a head high.
**9**	86	Adverse events of nightmare and vivid dreams	*nightmares cbd, vivid nightmares, vivid dreams, bad dreams, weird dreams, crazy dreams*	• idk ive been taking cbd oil to help me sleep and its giving me weird vivid dreams so luv that<br>• I've been taking a strong CBD oil recently before bed & it feels like I've graduated to a new level of sleep with immersive dreaming that's so vivid & sometimes weird. My dreams now also feel 10x longer then usual. Each night is a full-on experience now lol.<br>• Aside from the weird vivid dreams it gives me, CBD oil helps so sleep so gooood!
**10**	80	CBD legal status	*cbd legal, cannabis legal, weed legal, recommended cbd, but cannabis, use cbd*	• @twitter_user I am Canadian. We have three weed stores within walking distance to my home. I use cannabis oil to ease my back pain, and help me sleep. I am old. It is legal in Canada. And I hate murderous ignorant Putin so damn much!!<br>• @twitter_user Cbd helps me a whole lot with my sleep I am definitely happy its legal

				now..😎👍👍<br>• actually though why isn’t cbd legal in Iowa... I literally have to do like .5 mL of it just to comfortably sleep
**11**	77	CBD with CBN products	*sleep cbn, sleep cbd, cbd cbn, cbn cbd, cbn helps, also cbn*	• @twitter_user Cbd to give you the "entourage" effect, they work together better than alone, I find 1:1 helps me sleep the beat. CBN is also hitting the market which makes claims of superior sleep aid, didn\'t work for me but CBN did mute the mua le pain and cramps from a day of work<br>• @twitter_user CBN is better for sleep. Try to find a blend CBD/CBN<br>• @twitter_user There is a blend of CBD and CBN (CBN helps more with sleeping) called Dusk by @twitter_user and it’s the only thing that’s helped my insomnia
